# Truncated ring-A amaryllidaceae alkaloid modulates the host cell integrated stress response, exhibiting antiviral activity to HSV-1 and SARSCoV-2

**DOI:** 10.1038/s41598-023-28691-0

**Published:** 2023-01-30

**Authors:** James McNulty, Chanti Babu-Dokuburra, Jon Scattolon, Carlos Zepeda-Velazquez, Maribeth A. Wesesky, Jill K. Caldwell, Wenxiao Zheng, Jadranka Milosevic, Paul R. Kinchington, David C. Bloom, Vishwajit L. Nimgaonkar, Leonardo D’Aiuto

**Affiliations:** 1 Department of Chemistry & Chemical Biology, McMaster University, Hamilton, ON L8S 4M1 Canada; 2 Department of Psychiatry, Western Psychiatric Institute and Clinic, University of Pittsburgh School of Medicine, Pittsburgh, PA 15213 USA; 3 Department of Ophthalmology, University of Pittsburgh School of Medicine, Pittsburgh, PA USA; 4 Department of Molecular Microbiology and Genetics, University of Pittsburgh, Pittsburgh, PA USA; 5 Department of Molecular Genetics and Microbiology, College of Medicine, University of Florida, Gainesville, FL 32610 USA; 6 Second Xiangya Hospital, Xiangya School of Medicine, Central South University, Changsha, China; 7 Department of Biomedical Engineering, Carnegie Mellon University, Pittsburgh, PA USA; 8 Captis Diagnostics Inc, Pittsburgh, PA USA; 9 Veterans Administration Pittsburgh Healthcare System, 4100 Allequippa St (University Drive C), Pittsburgh, PA 15240 USA

**Keywords:** Natural product synthesis, Antivirals

## Abstract

The total synthesis of four novel mono-methoxy and hydroxyl substituted ring-A dihydronarciclasine derivatives enabled identification of the 7-hydroxyl derivative as a potent and selective antiviral agent targeting SARSCoV-2 and HSV-1. The concentration of this small molecule that inhibited HSV-1 infection by 50% (IC50), determined by using induced pluripotent stem cells (iPCS)-derived brain organ organoids generated from two iPCS lines, was estimated to be 0.504 µM and 0.209 µM. No significant reduction in organoid viability was observed at concentrations up to 50 mM. Genomic expression analyses revealed a significant effect on host-cell innate immunity, revealing activation of the integrated stress response via PERK kinase upregulation, phosphorylation of eukaryotic initiation factor 2α (eIF2α) and type I IFN, as factors potentiating multiple host-defense mechanisms against viral infection. Following infection of mouse eyes with HSV-1, treatment with the compound dramatically reduced HSV-1 shedding in vivo.

## Introduction

**Figure 1:**
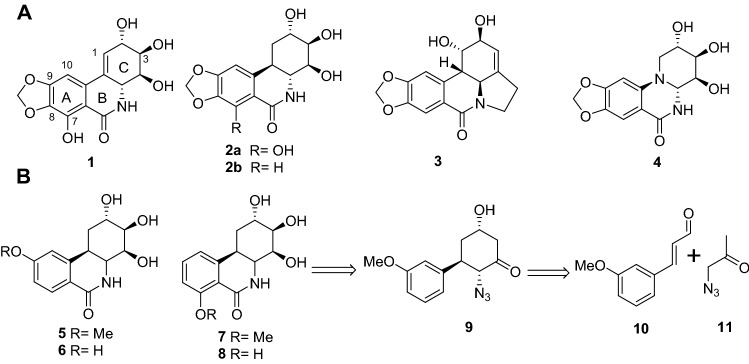
(**A**) Structure of natural amaryllidaceae constituents with potent antiviral activity, narciclasine (**1**), 1-deoxypancratistatin (*trans*-dihydronarciclasine) (**2a**), *trans*-dihydrolycoricidine (**2b**), lycorine (**3**) and the inactive synthetic 10b-aza analog (**4**). (**B**) The new trans-dihydronarciclasine analogs **5**–**8** and proposed retrosynthesis via the cyclohexane derivative **9**, which could be derived from cinnamaldehyde derivative **10** and azido acetone **11**.

Natural products continue to occupy a central role in drug discovery^1–4^, a tradition evidenced by the recent efforts in the identification of new leads against the SARSCoV-2-associated coronavirus^3^. Current approved and investigational therapeutics are either repurposed or designed agents that target key viral proteins such as protease or RNA polymerase inhibition^4^. Alkaloids isolated from various members of the amaryllidaceae plant family^5^ have wide pharmacological potential in view of the anticancer^6–16^, antiviral^17–22^ and acetylcholinesterase activities they have demonstrated. The antiviral activity of narciclasine (**1**) and a wide range of alkaloids (Fig. 1) of this class was reported three decades ago^17^. The potent antiviral activity of 1-deoxypancratistatin (**2a**) and *trans*-dihydrolycoricidine (**2b**)^23^ to DNA viruses such as Herpes (HSV-1, VZV) and RNA viruses including Zika (ZIKV) has also been recently reported^19,20,24^. The related alkaloid lycorine (**3**) has also been shown to significantly exhibit replication of the SARS-associated coronavirus^21^. The mechanism of action responsible for the spectrum of antiviral activity observed in the narciclasine class is not clear. Inhibition of protein biosynthesis through interaction with human eukaryotic translation elongation factor 1A (eEF1A) has been previously implicated and recently reviewed^22^. The broad-spectrum activity against not only DNA, but also against RNA viruses is intriguing and may imply the activation of a host-defense mechanism rather than a specific viral target. We envisioned that the elaboration of a potent and selective antiviral agent would thus serve as a valuable biological probe necessary to illuminate the mechanistic underpinnings responsible. Understanding this mechanism became a priority as its upregulation could provide a robust defense independent of rapidly mutating viral proteins, such as observed in SARSCoV2.

**Figure 2:**
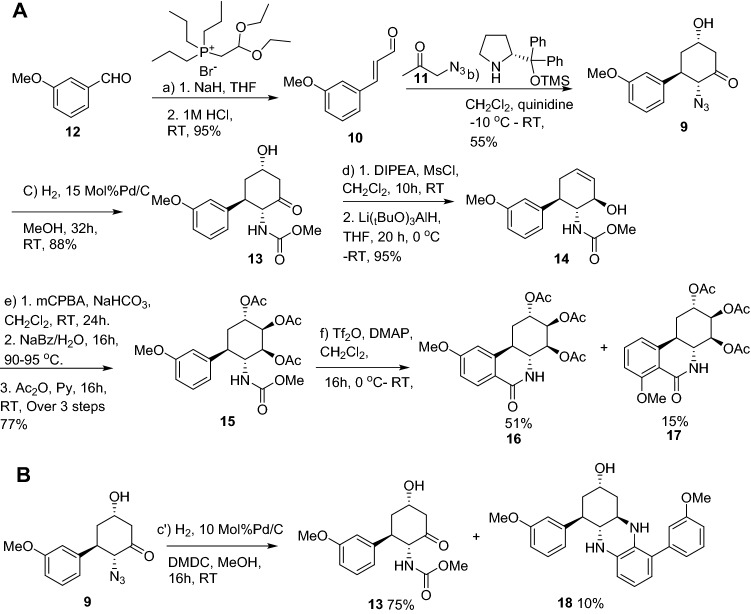
(**A**) Synthesis of ring-A truncated triacetates **16** and **17**: Pd/C loading and time (a) 1. (CH_3_CH_2_O)_2_CHCH_2_P(CH_2_CH_2_CH_3_)_3_Br, NaH, THF; 2. 1 M HCl, RT, 95%; (b) **11**, CH_2_Cl_2_, (*R*)-diphenylprolinol TMS ether, quinidine, − 10 °C—RT, 55%; (C) H_2_, 15 Mol%Pd/C MeOH, 32 h, RT, 88%; (d) 1. DIPEA, MsCl, CH_2_Cl_2_, 10 h, RT, 2. Li(*t*BuO)_3_AlH, THF, 20 h, 0 °C—RT, 95%; (e) 1. mCPBA, NaHCO_3,_ CH_2_Cl_2_, 24 h, RT; 2. NaBz/H_2_O, 16 h, 90–95 °C; 3. Ac_2_O, Py, 16 h, RT, over 3 steps 77%; (f) Tf_2_O, DMAP, CH_2_Cl_2_, 16 h, 0 °C—RT. (**B**) Formation of non-symmetrical dimer **18**: (C′) H_2_, 10 Mol% Pd/C, DMDC, MeOH, 16 h, RT.

Herein we describe the asymmetric synthesis of four new dihydronarciclasine analogs with ring-A mono-substituted C9-OMe (**5**), C9-OH (**6**), C7-OMe (**7**), and C7-OH (**8**), containing the fully functionalized ring-C (Fig. 2). Initial antiviral activity studies led to the identification of the 7-hydroxyl analog **8**, alone, as the structurally minimal analog demonstrating antiviral activity to HSV-1. Compound **8** was also found to exhibit potent and selective antiviral activity to the SARSCoV-2 coronavirus. We report initial findings on a biological target and mechanism of action of compound **8** consistent with the activity observed against both DNA and RNA viruses. We demonstrate that compound **8** activates the eukaryotic integrated stress response (ISR) and the resulting signaling network, inducing autophagy. Compound **8** also upregulates the sirtuin signaling pathway, with activation of innate immunity. Upregulation of these synergistic antiviral defenses in the host cell explains the broad-spectrum antiviral activity observed. Importantly, these processes are not dependent on interactions with highly mutable viral structural proteins and non-structural targets such as proteases, thus compound **8** represents an important lead towards advancing a stable, broad-spectrum antiviral agent. Compound **8** also demonstrated potent in vivo anti HSV-1 activity in a mouse model.

## Results

We have previously reported the total synthesis and antiviral studies of 1-deoxypancratistatin (**2a**) and *trans*-dihydrolycoricidine (**2b**)^23,24^. In this work, the synthesis of various modifications to the hydroxylated ring-C and their antiviral activities was investigated. For example, the C3-epimer of **2b**^25^, among other analogs, as well as the fully functionalized aza-analog **4**^26^ proved totally devoid of antiviral activity. To date, compound **2b** appears to be the minimal structure that exhibits potent antiviral activity, activity that is significantly enhanced by addition of the C7 hydroxyl group present in compound **2a**^17^. Further modifications to ring-C as a means of optimizing antiviral potency and selectivity further appear fruitless. These results turned our focus to the ring-A substituents as the last remaining fragment available for modification. All analogs investigated for RNA viral activity possess the 8,9-methylenedioxy substituent fused to ring-A^22^. Only one prior study has investigated alternative ring-A hydroxyl positionings. Alonso and co-worker reported the synthesis of the 8-hydroxyl analog, which was found to exhibit significantly reduced anticancer activity, though their antiviral activity was not investigated^27^. In order to delineate the contributions of a single hydroxyl or methoxy group substituent in ring-A to antiviral activity, we modified our original synthesis of 1-deoxypancratistatin (**2a**) and *trans*-dihydrolycoricidine (**2b**), starting from *meta*-anisaldehyde (**12**).

### Chemical synthesis

**Figure 3:**
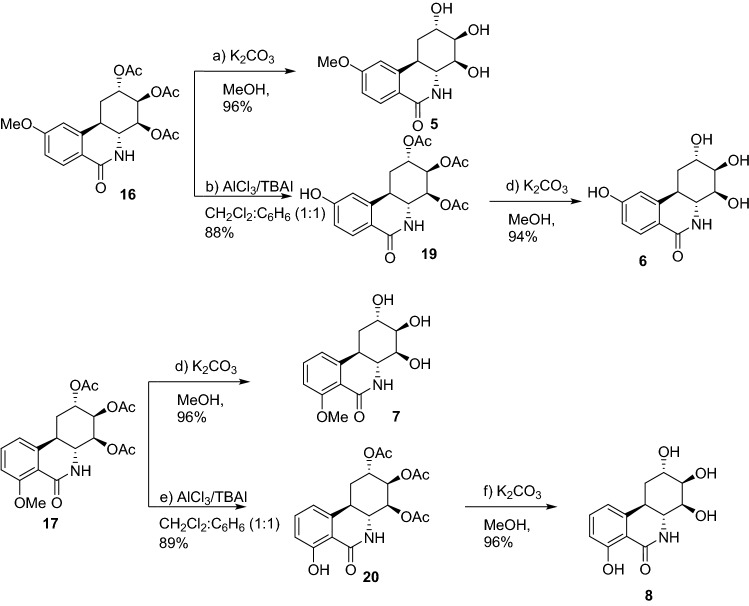
Synthesis of ring-A truncated *trans*-dihydronarciclasine derivatives (**5**–**8**). (a) K_2_CO_3_, MeOH, 95%; (b) AlCl_3_/TBAI, CH_2_Cl_2_:C_6_H_6_ (1:1) 88%; (C) K_2_CO_3_, MeOH, 94%; (d) K_2_CO_3_, MeOH, 96%; (e) AlCl_3_/TBAI, CH_2_Cl_2_:C_6_H_6_ (1:1) 89%; (f) K_2_CO_3_, MeOH, 96%.

The synthesis of the target compounds **5**–**8** was achieved by a convergent-divergent strategy via the common intermediate **15** (Fig. 2A) with significant modifications to our published synthetic protocol^23^. Commercially available 3-methoxybenzaldehyde **12** was subject to a two-carbon aldehyde to alkenal homologation using a diethyl acetal-functionalized Wittig reagent yielding the 3-methoxy cinnamaldehyde **10** in 95% yield. The iminium-ion mediated asymmetric organocatalytic stepwise [3 + 3] Michael-aldol sequence of **10** with azidoacetone **11** proceeded smoothly using the (*R*)-diphenylprolinol-trimethylsilyl ether and quinidine catalysts, providing the cycloadduct **9** in 55% isolated yield. Hydrogenation of the azido functional group of **9** with 10% Pd/C in the presence of dimethyldicarbonate gave the desired methoxycarbonyl protected cyclohexanone **13** as well as a minor side-product, which proved to be the structurally interesting mono-aromatized dimer **18** (Fig. 2B). To improve the selectivity favoring **13**, dilution (0.1 M) and increased reaction time allowed isolation of the methoxycarbonyl protected cyclohexanone **13** in 75% isolated yield along with the minor product **18** in 10% isolated yield. Interestingly, increased catalyst loading up to 15%, under otherwise identical reaction conditions, led only to the desired compound **13**. This minor side product indicates that the anticipated reduction of the azide **9** was successful, the free amino-ketone then undergoes an in-situ dimerization followed by dehydration and aromatization of one ring (perhaps via an enamine intermediate) producing **18**. The carbamate **13** and its enantiomer *ent*-**13**, prepared identically using (*S*)-diphenylprolinol-trimethylsilyl ether) were both found to be > 99% and > 98% enantiomeric excess (e.e.) respectively (baseline resolved) using chiral HPLC (AD-H column). Dehydration of **13** via the in-situ formed mesylate and immediate reduction of the ketone using the bulky lithium tri-tert-butoxyaluminium hydride yielded the equatorial alcohol **14** exclusively in 95% yield for the two steps. Epoxidation of the allylic alcohol **14** produces both diastereomeric epoxide which are opened stereospecifically through exclusive axial attack using sodium benzoate in water, yielding the 2,3-diaxial-4-equatorial triol, which was acetylated with Ac_2_O in the presence pyridine to give triacetate **15** in 77% overall yield from **13**. The phenanthridone ring was closed employing Banwell’s modification of the Bischler-Napieralski reaction^28^, giving the regioisomeric tricyclic products **16** and **17**. Compounds **16** and **17** were clearly resolved on silica and separated by gradient elution flash chromatography starting from 100% dichloromethane, yielding **16** (major) and **17** (minor) in 51% and 15% isolated yields respectively. Selective cleavage of the C9-methyl ether in **16** proved problematic. Attempted cleavage using standard reagents such as TMSI or BBr_3_ gave mixtures of products, and the use of BCl_3_-dimethylsulfide resulted in ester cleavage. Nevertheless, it was discovered that the use of AlCl_3_ in the presence of the phase transfer catalyst tetrabutylammonium iodide (1:3) was successful, following a protocol developed by Steglich and co-workers^29^. This procedure provided phenolic compound **19** in 88% yield. The same reaction conditions were repeated for the cleavage of C7-methyl ether **17**, which also provided the demethylated phenol **20** in 89% yield. Global saponification of the acetate groups was readily achieved in compounds **16**, **19**, **17** and **20** using K_2_CO_3_ and aqueous methanol finally yielding the required compounds **5**,** 6**, **7**, and **8** respectively (Fig. 3).

### Antiviral activity

We recently showed that *trans*-dihydrolycoricidine (**2b**) inhibits HSV-1 replication more efficaciously than acyclovir (ACV), and prevents HSV-1 reactivation from latency in an ex vivo mouse model^20^^,^^25^. Also, **2b** was shown to exhibit moderate potency against HSV-1 strains that have been reported to be resistant to ACV, such as the tk- strain of HSV-1 that lacks thymidine kinase activity^30^, and the PAAv strain that has developed mutations in viral DNA polymerase following incubation with phosphonoacetic acid^31^. The antiviral activity of **2b** against other DNA viruses (HSV-1, HCMV, HBV), and RNA viruses (HCV, ZIKV strains FSS-13025 and PE-243) was also demonstrated^20^. Slightly higher toxicity was observed for **2b** in comparison to acyclovir in fibroblasts and hepatocytes, while a lower toxicity was noted in iPSC-derived neural progenitor cells (NPCs).

### C7-Monohydroxyl analog **8** exhibits anti-HSV-1 activity

**Figure 4:**
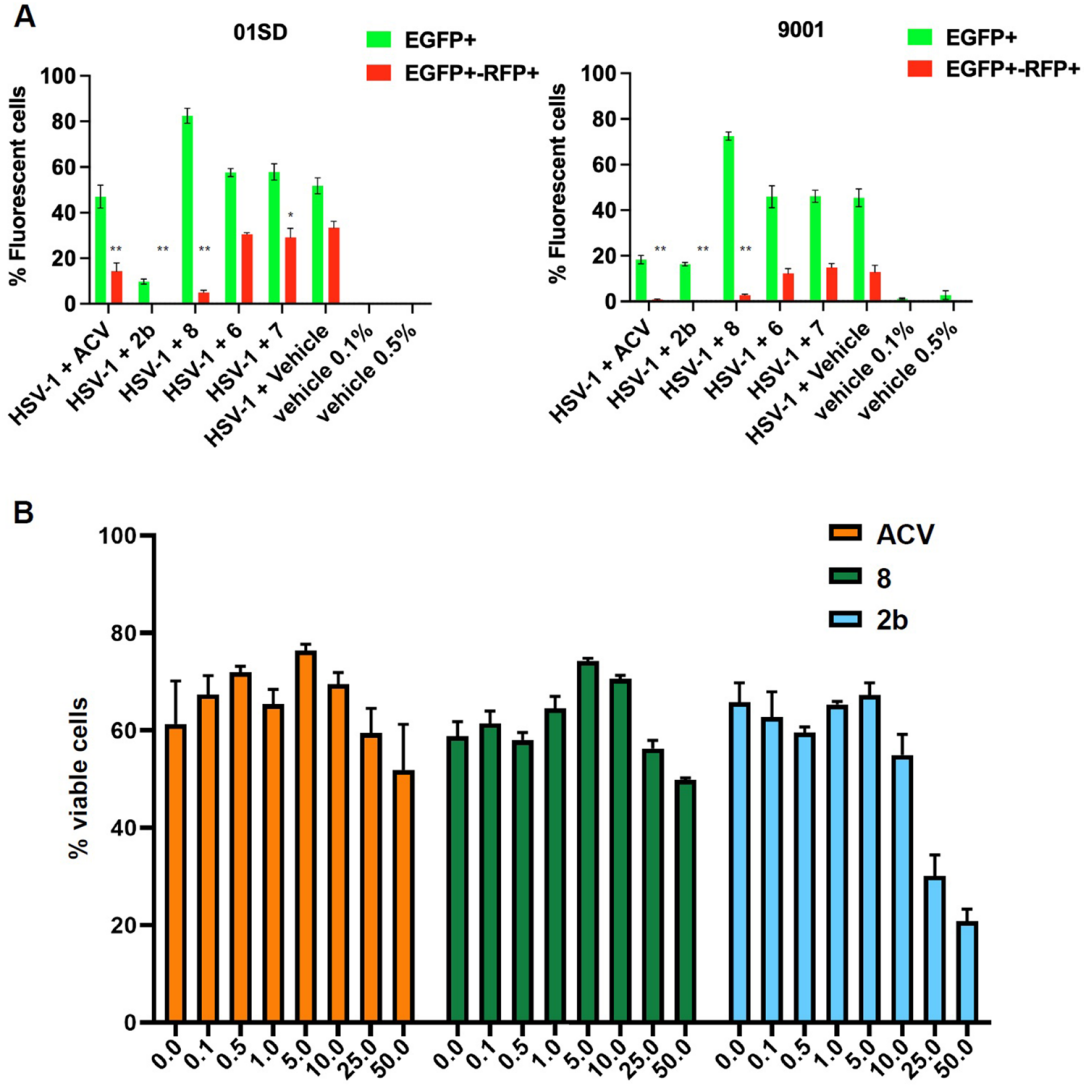
Compounds 2b and 8 inhibit HSV-1 in human iPSC-derived NPCs to different extents. (**A**) Flow cytometry (FC) analysis of uninfected and infected NPC lines 01SD and 9001 in the presence or absence of ACV, **2b** and its derivatives **6**, **7** and **8** using an engineered HSV-1 construct DualF, expressing EGFP from an IE gene promoter and RFP from a late gene promoter. NPCs were infected at a multiplicity of infection of 0.1. The aforementioned compounds were added at two hours p.i., and cultures were analyzed at day 3 p.i.. The fractions of cells showing fluorescence of the appropriate color are indicated. (**B**) Cytotoxicity of **2b**, **8** and ACV at varying concentrations in uninfected 01SD NPC cultures was assessed by FC using fixable viability dye. The data represent an average of six independent experiments. Error bars represent standard deviations. One-way analysis of variance (ANOVA) followed by Bonferroni post-hoc test was used to compare the reduction of EGFP-RFP^+^ cells after treatment of infected cells with tested compounds when compared to HSV-1 + vehicle: *P = 0.0124; **P < 0.0001.

The antiviral activity of the new mono-hydroxyl and methoxy derivatives **5**, **6**, **7**, and **8** derivatives was initially tested in two neural precursor cell (NPC) lines and compared with acyclovir, the “gold standard” treatment for HSV infections. NPCs were utilized in this initial screen for two reasons; (i) the need to employ a platform composed of CNS cells, and (ii) the higher susceptibility of NPCs to HSV-1 when compared to neurons. Human NPCs were derived from iPSCs as previously described^32^. NPCs were infected with an HSV-1 construct (DualF) expressing enhanced green fluorescent protein (EGFP) and monomeric red fluorescent protein (RFP) under the control of the viral promoters ICP0 and Glycoprotein C, respectively^32^, at a multiplicity of infection** (**MOI) of 0.1, as detailed in the Experimental section B. The expression of the fluorescent reporter genes EGFP and RFP were considered as proxies of viral gene expression. Two hours after the infection the inocula were removed and cells were cultured in the presence of the above described monomethoxy/hydroxy analogs at the concentration of 10 μM or ACV at 50 μM and assayed at day 3 post infection (p.i.). Flow cytometry (FC) analysis indicated that compound **8** reduced the percentage of EGFP^+^-RFP^+^ cells by 6.76- and 4.65-fold and in 01SD and 9001 NPCs, respectively, when compared to infected cells exposed to the vehicle (Fig. 4A). Compound **2b** resulted in the greatest reduction of fluorescent cells (01SD: (8016-fold; 9001: 424-fold). Surprisingly, no anti-HSV-1 activity was exhibited by analogs **5**, **6** or **7** (Fig. 4A). Compound **5** also proved incompletely soluble in DMSO and was therefore not considered further. At concentrations up to 50 μM, the cell viability of ACV and compound **8** treated cultures were comparable (Fig. 4B). Conversely, a reduction in cell viability was observed in **2b**-treated cultures starting from 25 μM in accord with the cytotoxicity previously discussed for **2b**. Once again, no anti-HSV-1 activity was exhibited by compounds **6** and** 7**. Thus, quantification of viral infection and cell viability based structural-activity relationship analysis using monolayer 2D NPC cultures identified compound **8** as a novel anti-HSV-1 drug with an efficacy comparable or superior to ACV. *The antiviral potency of 2b and 8, and selectivity demonstrated by 8 alone, reveals a privileged pharmacophore in compound 8, unique in the further refinement of the selective antiviral activity that is observed.*

### The anti-HSV-1 activity of **8** is comparable to **2b** in brain organoids

**Figure 5:**
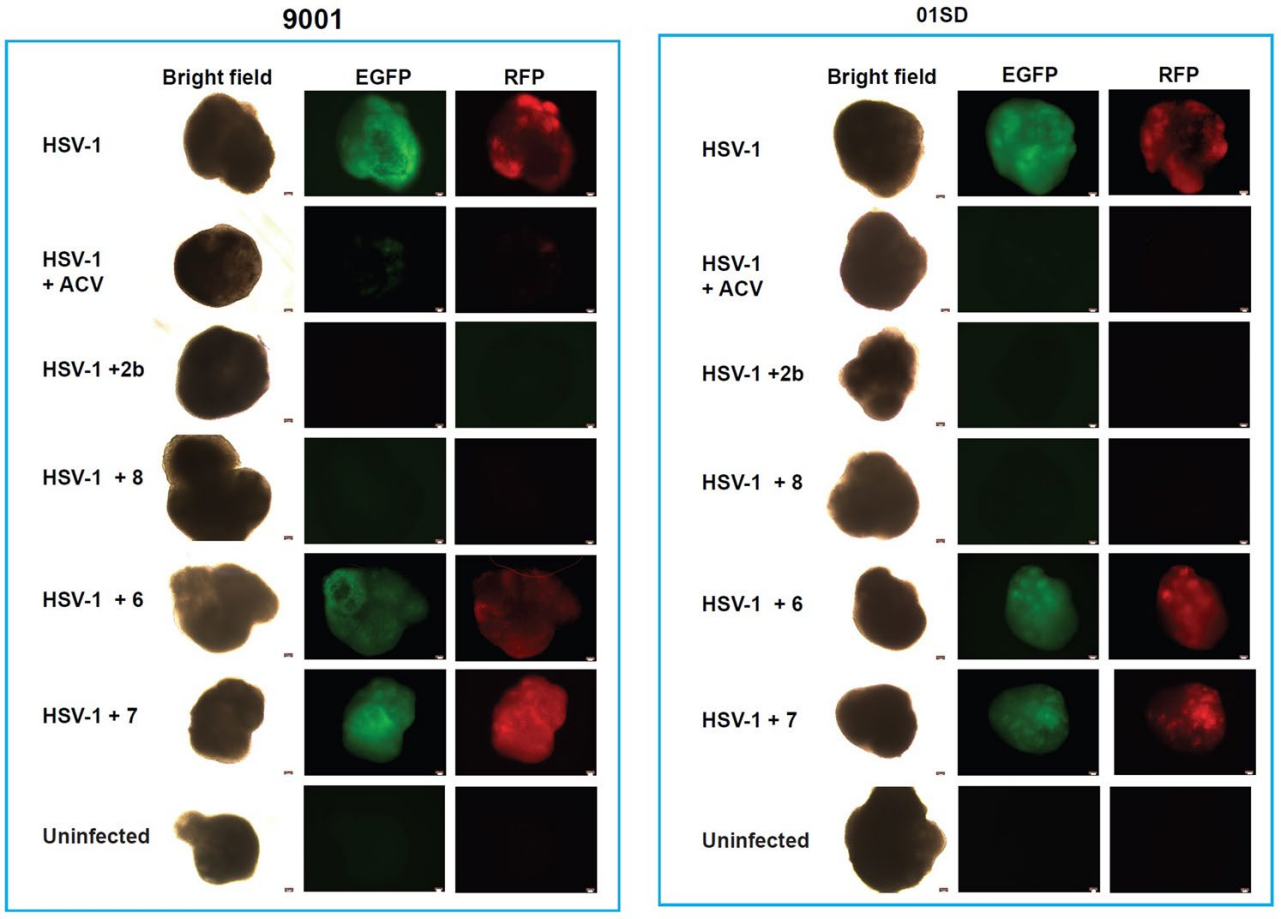
The anti-HSV-1 efficacy of compound **8** is comparable to **2b** and ACV, in human brain organoids. Fourteen-week old brain organoids generated from 9001 and 01SD iPSC-lines were infected individually with HSV-1 DualF construct (6000 pfu/organoid). Two hours after infection inocula were removed, organoids washed and cultured in the presence of the tested compounds. The expression of the fluorescent reported genes EGFP and RFP was visually inspected under a fluorescent microscope at day 3 p.i. N = 3 for each condition. Scale bar: 50 μm.

Although the classical 2D monolayer cultures facilitate drug screening processes, they do not recapitulate the 3D architecture and the physiological microenvironment of a living tissue. conditions. As a consequence, drug effects in a 2D microenvironment may not accurately reflect drug effects in vivo. There is increasing evidence that three-dimensional cultures increase the predictability of drug activity in vivo^33^. Thus, the inhibitory activity of **2b** and its derivatives against HSV-1 was investigated in a 3D culture platform consisting of brain organoids. A characterization of differentiating organoids is depicted in Supplementary Fig. 1. Fourteen-week old brain organoids generated from 9001 and 01SD iPSCs were infected individually for 2 h with HSV-1 DualF virus (6000 pfu/organoid). After the infection the organoids were cultured in the presence or absence of tested compounds (10 μM) or ACV (50 μM). At day 3 p.i. no expression of EGFP and RFP reporter genes was observed in **2b**- and **8**-treated infected 9001 and 01SD organoids. As expected, ACV also efficiently inhibited HSV-1 infection (Fig. 5). Conversely, no anti-HSV-1 activity was exhibited by derivatives **6** and **7** (Fig. 5). These results confirm the ability of the new analog **8** alone to inhibit HSV-1 infection.

**Figure 6:**
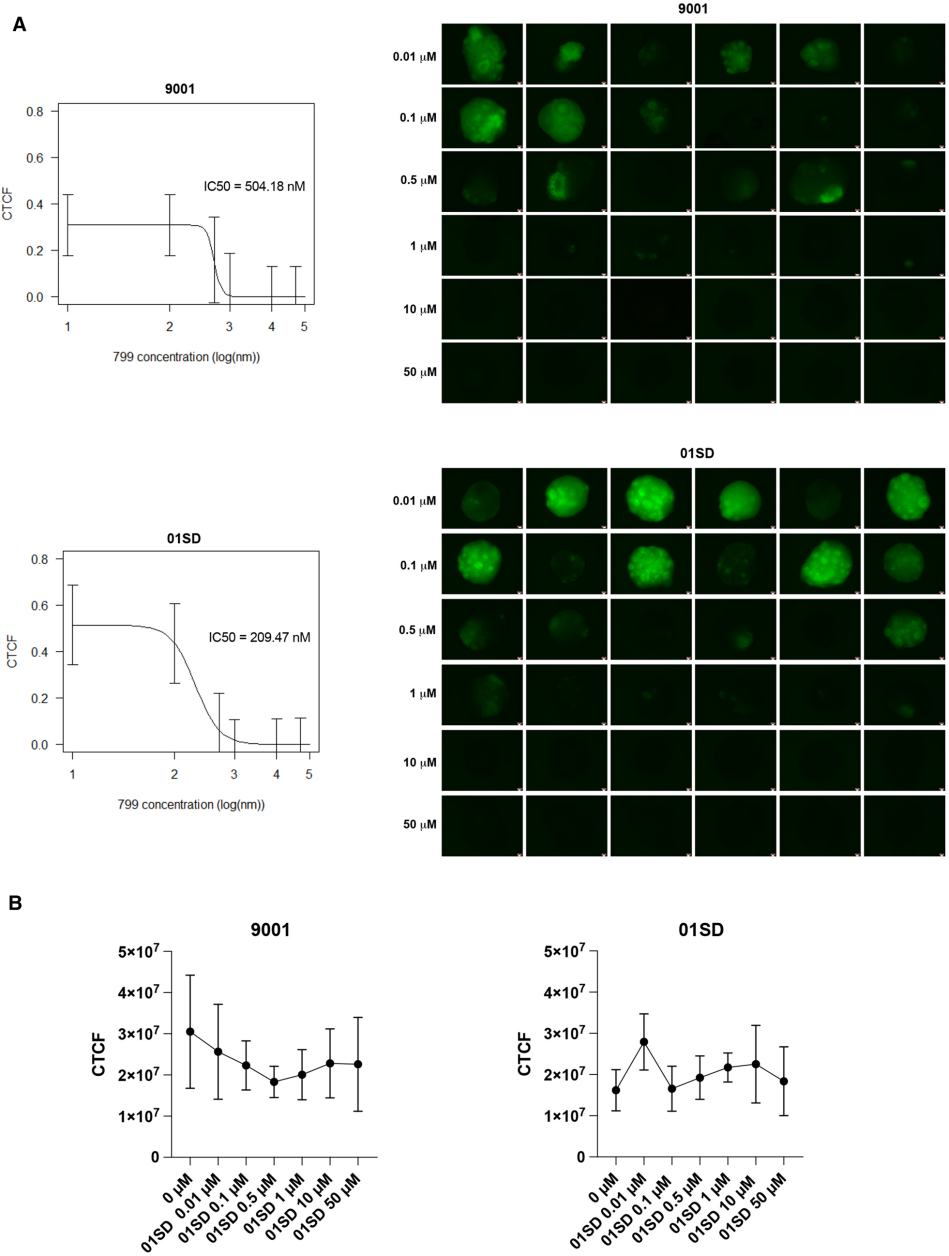
Antiviral efficacy and cytotoxicity of 8 against HSV-1 in human brain organoids. (**A**) Sixteen-week old 9001 and 01SD brain organoids were infected individually with HSV-1 DualF construct (6000 pfu/organoid). After 2 h the inocula were removed and **8** (799) was added to varying concentrations (100 nM–50 μM). The IC50 was estimated by determining the corrected total cell fluorescence (CTCF) obtained by measuring EGFP fluorescence in organoids using ImageJ and normalizing for background using equation CTCF = integrated density − (area of the organoid × mean fluorescence of background readings). (**B**) Cytotoxicity of **8** without HSV-1 infection was assessed using calcein-AM. The fluorescence of calcein-AM in each organoid was measured and CTCF was determined as described above. The data represent an average of six independent experiments. Error bars represent standard deviations. One-way analysis of variance (ANOVA) followed by post hoc test using Tukey multiple comparison correction was used to compare cell viability at different drug concentrations No significant differences in cell viability were found between conditions. Scale bar: 50 μm.

Next, the concentration of **8** that inhibited HSV-1 infection by 50% (IC50) in 9001 and 01SD organoids was determined. Sixteen-week old brain organoids were infected as described above and exposed after 2 h to increasing **8** concentration ranging from 0.01 to 50 μM. At day 3 p.i. the IC50 of **8** was determined by measuring the intensity of the fluorescent reporter gene EGFP (whose expression is under the control of promoter of the immediate early HSV-1 gene ICP0) in the infected organoids was investigated. The EGFP expression was used as a proxy for viral gene expression during early stages of HSV-1 acute infection. The IC50 of compound **8** was determined to be 0.504 µM in 9001 and 0.209 µM in 01SD organoids (Fig. 6A). No significant reduction in organoid viability, determined by utilizing calcein AM, was observed at concentrations up to 50 μM, indicating a wide margin of safety in this assay (Fig. 6B).

### Compounds **2b** and **8** exhibit potent antiviral activity in the NIH-NIAID in vitro SARS-CoV2 viral replication assay

The antiviral activity of compounds **2b** and **8** to the COVID-19 associated coronavirus SARS-CoV2 in Calu3 (ATCC, HTB-55) cells was assessed independently at the National Institutes of Health (NIH). Compound **2b** was determined to exhibit an IC50 of 0.18 µM and CC50 of 3.48 µM, giving a therapeutic selectivity of 19. The new analog **8** proved to be both more potent and slightly better selectivity, exhibiting an IC50 of 0.09 µM and CC50 of 1.92 µM, giving a therapeutic selectivity of 20. The positive control remdesivir exhibited an IC50 value of 0.06 µM in this assay (supplementary Fig. 3).

### RNA-Seq analysis demonstrates compounds **2b** and **8** activate an integrated stress response

Given the activity observed to both DNA and RNA viruses, we performed RNA sequencing analysis on host cells treated with the synthetic alkaloids in order to assess changes in transcription levels with the goal of illuminating operative pathways. Uninfected 02SF NPCs cells were incubated with **2b**, **8**, **6** (802), or **7** (718), (10 μM) for 72 h. Following extraction, host RNA sequences were analyzed. A total of 12,433 human genes were expressed at TPM ≥ 5 in at least one replicate of untreated NPCs. When NPCs were incubated with compounds **2b**, **8**, **6**, or **7**, the expression of 7766 (62.5%), 7433 (59.8%), 256 (2.1%), and 33 (0.3%) host cell genes, respectively, were significantly altered (FDR corrected p ≤ 0.05, maximum group mean expression TPM ≥ 5). Using quantitative RT-PCR, we confirmed alterations in mRNA levels for four selected genes that were significantly altered by compounds **2b** and **8** in the RNA sequencing analyses (Supplementary Fig. 2).

**Figure 7:**
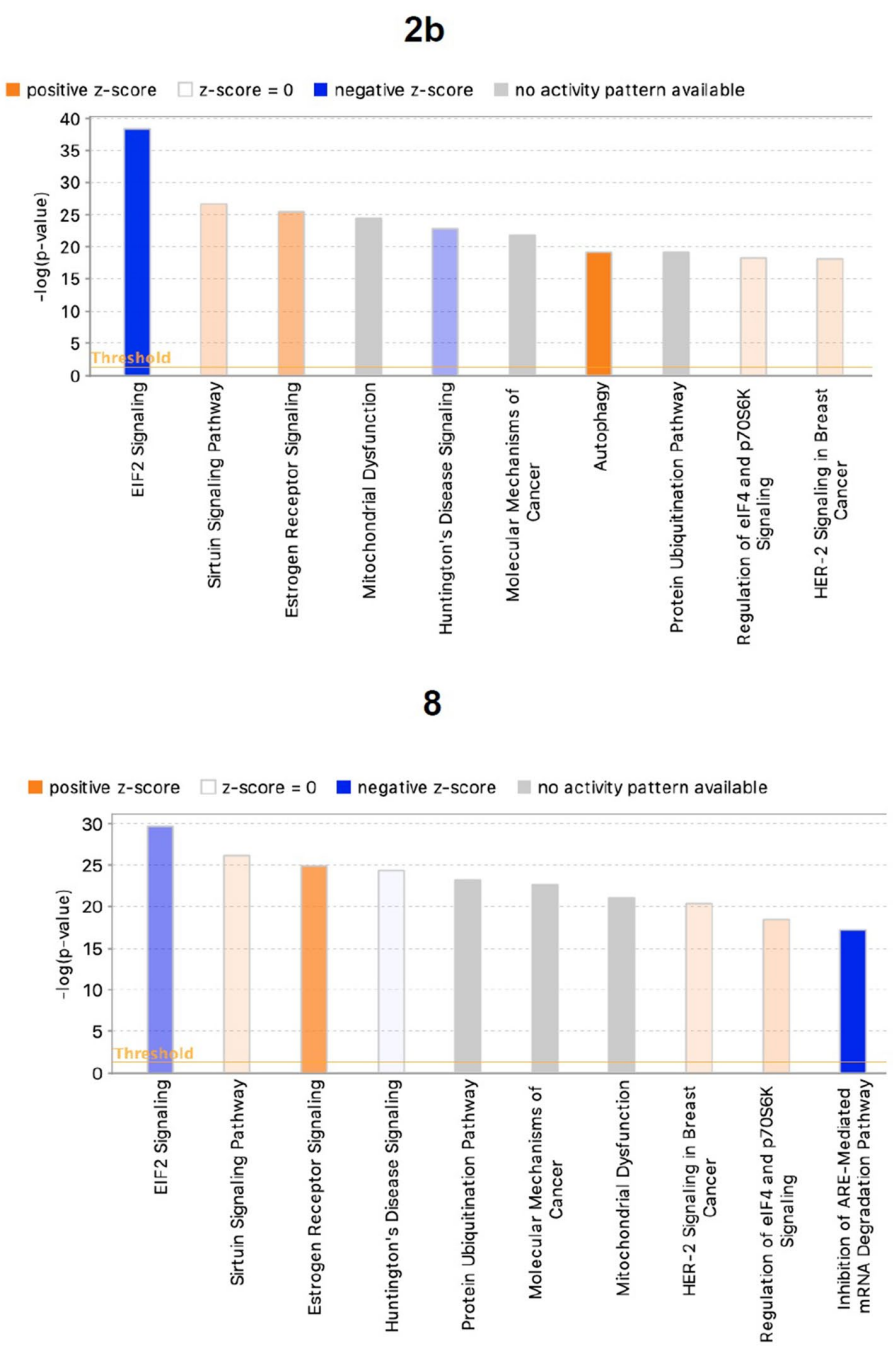
Human gene pathways significantly altered by **2b** and **8**. Differentially Expressed Genes (DEG) measured by comparing **2b**- and **8**-treated NPCs vs. vehicle-treated NPCs were subjected to Ingenuity Pathway Analysis (IPA). The top 10 pathways showing the greatest statistical significance (Fisher’s Exact Test p-value) are shown. Activation (+ve z-score, orange bars) or inhibition (−ve z-score, blue bars) of each pathway is a measure of experimentally determined gene expression changes reported in the literature. The intensity of color indicates the degree of activation/inhibition. Pathways without an activity pattern are in grey. Ratios (orange square above each pathway) represent the degree of overlap between DEG and all members of a given pathway. Y-axis on the right indicates ratio values.

Ingenuity Pathway Analysis (IPA) identified canonical pathways that were significantly altered when hiPSC-NPCs were incubated with **2b** and **8**. Statistical significance was calculated using right-tailed Fisher Exact Probability Tests; biological pathways showing p-value < 0.05 were considered statistically significant. The ten most significantly altered pathways are shown in Fig. 7. The eukaryotic initiation factor 2 (EIF2) signaling pathway was most significantly altered (**2b**: z score: − 2.857, p-value = 5.09E−39; **8**: z score: − 1.763, p-value = 2.43E−30). IPA analyses also indicated net upregulation in the autophagy and sirtuin signaling pathways.

**Figure 8:**
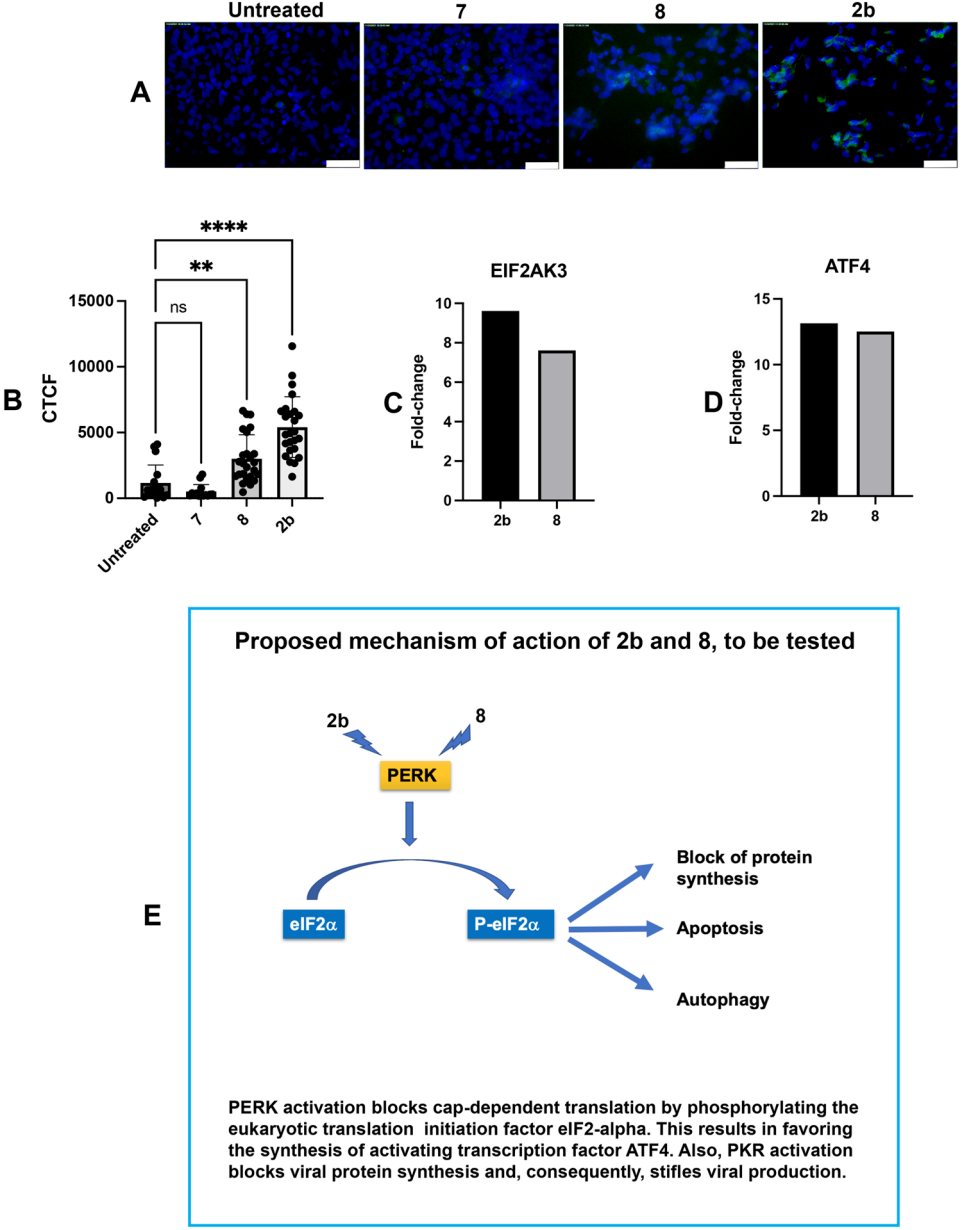
Activation of the integrated stress response in 2b- and 8-treated NPCs. (**A**) NPCs derived from 9001 iPSC lines were exposed to compounds **7**, **8**, and **2b** (10 μM). After 24 h, cells were fixed and stained with an antibody that detects eIF2α phosphorylated on Serine 51 (EIF2S1). Nuclei were counterstained with Hoechst 33342, and phosphorylation was assessed by fluorescence microscopy. (**B**) The corrected total cell fluorescence (CTCF) was obtained by measuring EIF2S1 fluorescence in cells where a fluorescent signal could be detected and normalizing for cellular area using equation CTCF = integrated density − (cellular area × mean fluorescence of background readings). Error bars are standard deviations. One-way analysis of variance (ANOVA) followed by Bonferroni post-hoc test was used to compare CTCF among the different conditions: ** (*P* = 0.0042); **** (*P* < 0.0001). (**C**,**D**) Up-regulation of the EIF2A kinase EIF2AK3 (**C**) and the activated transcription factor 4 (ATF4) (**D**) in **2b**- and **8**-treated NPCs. Fold-changes were calculated from RNA-seq data described in the methods section. (**E**) Proposed mechanism of action of **2b** and **8**.

These results immediately suggested the hypothesis that compounds **2b** and **8** may affect viral replication by triggering the integrated stress response (ISR), which, in turn, could block 5’ methyl cap-dependent mRNA translation^34,35^. To test this hypothesis, we investigated the phosphorylation of the α subunit of eukaryotic initiation factor 2 (eIF2α ~ P) on serine 51 in NPC cultures exposed to compounds **2b** and **8**, and compared to untreated NPCs or NPCs treated with the derivative **7**, that does not exhibit any antiviral activity. Indeed, immunocytochemistry analysis demonstrated increased eIF2α ~ P immunoreactivity in the **2b**- and **8**-treated cultures (Fig. 8). These results confirm that phosphorylation of e1F2α is upregulated and that activation of the integrated stress response (ISR) is one of the main mechanisms underlying the antiviral activity of these small molecules. RNAseq analysis also showed that phosphorylation of the α subunit of eukaryotic initiation factor 2 is mediated by the eIF2α kinase EIF2KA3 (PKR-ER or PERK) in the **2b**- and **8**-treated cultures (Fig. 8). The RNA sequencing data is thus fully consistent with the hypothesis that **2b** and **8** trigger a host antiviral response by activating an ISR.

ISR acts at the level of translation initiation to suppress global protein synthesis (while enhancing translation of several proteins involved in cellular recovery). ISR-induced downregulation of virus protein synthesis is an effective antiviral defense mechanism. Furthermore, eIF2- phosphorylation leads to increased synthesis of activating transcription factor 4 (ATF4), which is then transported to the nucleus to transactivate genes necessary for the cell to adapt the stress such as autophagy^36^. In HSV-1-infected cells. autophagy proteins target viral components or virions for lysosomal degradtion^37^. Furthermore, the activation of the sirtuin pathway reinforces the antiviral properties. Interestingly, SIRT1, a NAD-dependent deacylase/mono-ADP ribosyltransferase that can inhibit the growth of DNA and RNA viruses was shown to be up-regulated (Supplementary Fig. 2). The upregulation of SIRT1 may also contribute to the broad-spectrum antiviral activity observed with compounds **2b** and **8**^38,39^.

### Compound **8** exhibits in vivo anti HSV-1 activity in a mouse model

**Figure 9:**
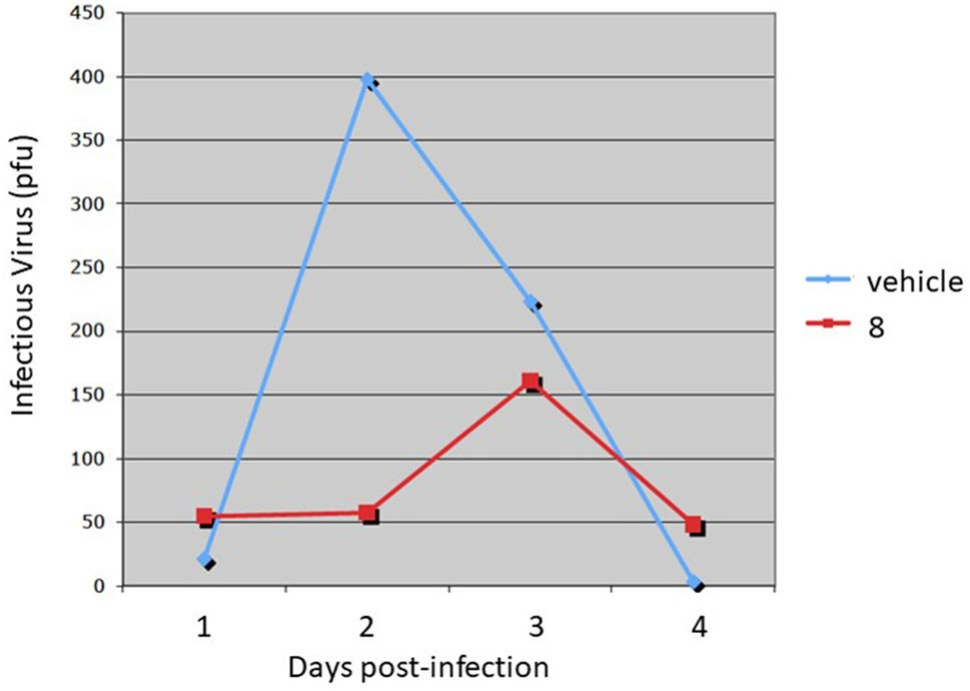
In vivo antiviral activity. ND4 swiss mice were infected by corneal scarification with 1 × 10^5^ pfu of HSV-1 strain 17syn+. Eyes were swabbed daily for 4 days and assayed for infectious virus by standard plaque assay. Compound **8** (code R799) dramatically reduces HSV-1 shedding in the mouse eye.

The antiviral efficacy of compound **8** was further investigated and confirmed in an in vivo mouse model. ND4 Swiss mice were infected in the eyes with HSV-1 following light corneal scarification as described in the Materials and Methods section. At the same time that the virus was applied, one group of mice (n = 10) were treated a 50uM solution of compound **8** in DMSO and the other group (n = 10) were treated DMSO (vehicle). The eyes were treated with either vehicle or compound **8** each day for 4 days. The eyes were swabbed daily and infectious virus shedding was assessed by standard plaque assay. As shown in Fig. 9, the eyes treated with compound **8** dramatically reduced the yield of infectious virus in the tears compared to vehicle treated eyes. The titer of infectious virus in the vehicle treated eyes peaked at day 2 post-infection, whereas the peak was delayed by one day in the **8** treatment group, being reduced almost threefold.

## Conclusions

In conclusion, we report the development and antiviral activity of a novel truncated ring-A synthetic alkaloid **8** against both HSV-1 and SARSCoV2. Mechanistic investigations demonstrate activation of host defense through upregulation of the integrated stress response pathway resulting in autophagy. This work demonstrates the application of a small molecule antiviral agent to induce innate immunity through activation of the EIF2 kinase (EIF2AK3 or PERK) as a mechanism for upregulation of the ISR. Upregulation of host-cell defense machinery following viral infection with a therapeutic prototype such as **8** reveals an attractive paradigm for the development of a broad-spectrum antiviral agent with predicted lower propensity for antiviral drug resistance. The ability to activate innate antiviral immunity through type I and/or III interferon stimulation has been described for small molecule lipids such as bile acids^40^, retinoids^41^, and the topical antiviral agent imiquimod^42^, involving complex regulatory pathways. Prior to this work, the contribution of the ring-A oxy-substituents of narciclasine-type alkaloids to antiviral activity was unknown^22^. While the ability of this class of alkaloid to inhibit protein biosynthesis at the ribosomal level was known, the target and mechanism was not clear^22^. Previous studies have implicated inhibition of host cell protein biosynthesis through translation elongation factor 1A. The complex marine ascidian derived depsipeptide plitidepsin has been shown to have anti SARSCoV2 activity via inhibition of elongation factor eEF1A^43^. The present work indicates that the truncated narciclasine-type alkaloids also act significantly upstream of this, and that inhibition of translation is in part a consequential event mediated through P-eIF2-alpha. The RNA sequencing profile provides a molecular signature that allows development of a selective antiviral agent, as prolonged and over activation of the ISR can lead to programmed cell death. This work identifies upregulated phosphorylation of host eIF2α as a precise target consistent with the known biological activity of these alkaloids. Modulated activation of the ISR may then lead to decreased protein expression and autophagy, resulting in potent antiviral activity in a selective manner.

## Methods

### Solvents and reagents

Chemicals and solvents were purchased from Acros, Aldrich, J.T. Baker, Caldeon, Solvay and Fluka and used as received with the exceptions below. Deuterated solvents were obtained from ACP Chemicals, Toronto, Canada. Tetrahydrofuran (THF), diethyl ether (Et_2_O), and toluene were distilled from sodium/benzophenone under an atmosphere of dry nitrogen; dichloromethane (CH_2_Cl_2_) was distilled from calcium hydride under an atmosphere of dry nitrogen; methanol (MeOH) was distilled from magnesium turnings under an atmosphere of dry nitrogen; triethylamine (NEt_3_), *N*,*N*-diisopropylethylamine (Hünig’s base) and pyridine were distilled from potassium hydroxide under an atmosphere of dry nitrogen; solid sodium hydride (NaH) was obtained by filtration and washing with *n*-hexanes.

### Reaction handling

All non-aqueous reactions were performed in flame dried round bottom flasks or in non-flame-dried amber 1.5-dram vials. Reactions were magnetically stirred and monitored by thin-layer chromatography (TLC) unless otherwise noted. TLC was performed on Macherey–Nagel silica gel 60 F_254_ TLC aluminum plates and visualized with UV fluorescence quenching and potassium permanganate (KMnO_4_) or 2,4-dinitrophenylhydrazine or *p*-anisaldehyde stains^1^. Concentrations under reduced pressure were performed by rotary evaporation at 40 °C at the appropriate pressure unless otherwise noted. Column chromatographic purification was performed as flash column chromatography with 0.3–0.5 bar pressure using Silicycle silica gel (40–63, 60 Å) or EcoChrom silica gel (12–26, 60 Å). Distilled technical grade solvents were employed. The yields given refer to chromatographically purified and spectroscopically pure compounds unless stated otherwise.

### Nuclear magnetic resonance (NMR) spectroscopy

^1^H, ^13^C{1H}, DEPTq, COSY, HSQC, and HMBC NMR spectra were obtained on Bruker DRX-500, AV-600, and AV-700 spectrometers. All ^1^H NMR spectra were referenced relative to SiMe_4_ through a resonance of the employed deuterated solvent or impurity of the solvent; chloroform (7.26 ppm), DMSO (3.33 ppm) and methanol (3.31 ppm) for ^1^H NMR; chloroform (77.00 ppm), DMSO (39.52 ppm) and methanol (49.00 ppm) for ^13^C NMR. All NMR spectra were obtained at RT (*ca*. 22 °C) unless otherwise specified. The data is reported as (s = singlet, d = doublet, t = triplet, m = multiplet or unresolved, br = broad signal, coupling constant(s) in Hz, integration). ^13^C-NMR spectra were recorded with complete ^1^H-decoupling. Service measurements were performed by the NMR service team of the Nuclear Magnetic Resonance Facility at McMaster University by Dr. Bob Berno and Dr. Hilary A. Jenkins.

### Mass spectrometry

Mass spectrometric analyses were performed as high-resolution ESI measurements on a Waters/Micromass QTof Global Ultima (quadrupole time-of-flight mass spectrometer) or high-resolution EI in a Waters/Micromass GCT (time-of-flight mass spectrometer) instrument by the mass spectrometry service of the McMaster Regional Centre for Mass Spectrometry (MRCMS) at McMaster University by Megan Fair and Leah Allan under the supervision of Dr. Kirk Green.

### Enantiomeric ratios

Enantiomeric ratios were determined using an Agilent 1220 Infinity HPLC manual injection with a variable wavelength detector, using a Daicel Chiralpak^®^ AD-H column (150 × 4.6 mm, 5µ), n-hexane/iPrOH (80:20) as a mobile phase; flow rate 0.75 ml/min, column temperature 25 °C, λ236 nm, sample 1 mg / 1 ml dissolved in the mobile phase.

### Optical rotations

Optical rotations were measured on a Perkin-Elmer 241 MC polarimeter, [*α*] is given in degcm^3^g^−1^ dm^−1^ and *c* is given in g cm^−3^.

### Cell lines

Human iPSC (hiPSC) lines subclone alias R139361416_SD and CR00427420_SA; RUCDR Infinite Biologics; Piscataway, New Jersey, United States) were employed in this study. The iPSC lines R139361416_SD and CR00427420_SA were denoted as 01SD and 9001, respectively, in this study.

### Generation of neural progenitor cells (NPCs)

Human iPSC cells (hiPSCs) were obtained from the Rutger’s University Cell & DNA Repository (Piscataway, NJ, USA). No human participants/data/samples were used in the current study. All samples are from a public repository (RUCDR) and are deidentified. HiPSCs were cultured in mTeSR1-plus medium supplemented with dual SMAD inhibitors SB431542 (10 µM) and LDN193189 (100 nM) (NPS/Dual-SMAD) to induce neuroectoderm formation. After 8–10 days, neural rosettes were manually isolated, transferred into Matrigel coated plates and cultured in StemDiff Neural Progenitor Medium (STEMCELL Technologies) for the expansion of NPCs.

### Generation of organoids from brain organoids

Human iPSC (hiPSC) lines 01SD and 9001 were employed to generate brain-like organoids. Organoids were generated as previously described (ref) with some modification in the initial part regarding the generation of spheroids containing neural rosettes. hiPSCs cultured with mTeSR™ plus medium (STEMCELL Technologies) in Matrigel-coated 6-well plates were detached with Accutase and then dissociated into single cell suspension by gently pipetting. They were seeded into low attachment U-bottom 96-well plates at the density of 9000 cells/well, in mTeSR plus medium supplemented with Rho-associated protein kinase inhibitor (ROCK inhibitor) Y27632 (STEMCELL™) to generate embryoid bodies (EBs). After three days, to induce neuroectoderm, the medium was switched to Essential 6 medium (ThermoFisher Scientific) supplemented with dual SMAD inhibitors SB431542 10 µM (MilliporeSigma S4317-5MG) and LDN193189 100 nM (MilliporeSigma SML055-9-25MG. Cultures were observed on daily basis.

On day 8, differentiating EBs were rinsed with Dulbecco's Modified Eagle Medium: Nutrient Mixture F-12 (DMEM/F12, Gibco 11330-032) and cultured in neuronal medium (DMEM/F12 supplemented with 1X MEM Nonessential Amino Acid supplement (MEM-NEAA, CORNING^®^ 25–025-CI), 1X Glutamax (Gibco 35050-061), 1 × N_2_ supplement (Gibco 17502-048) and 1 µg/mL Heparin (STEMCELL™ 07980)) in untreated 10-cm petri dishes. These plates were placed on an orbital shaker in the incubator at 70 rpm (Orbi-Blotter™). Culture medium was halved every three days.

On day 20, the culture medium was replaced with cortical organoid differentiation medium I ((CODMI: DMEM:F12/Neurobasal (1:1 v/v) supplemented with 1X Glutamax, 1X B-27 (VitA[-]), 0.5X Non-essential amino acids, 0.5X N-2, Insulin (2.5 μg) and 1X penicillin–streptomycin (P/S). On day 25, CODM-I medium was replaced with cortical organoid differentiation medium II (CODM-II: DMEM/F12-Neurobasal (1:1 v/v) supplemented with 1X glutamax, 1X B-27 (VitA[+]), 0.5X Non-essential amino acids, 0.5X N-2, Insulin (2.5 μg), BDNF (10 ng/mL), and 1X P/S. Culture medium was changed every 3 days. On day 42, CODM-II medium was replaced with BrainPhys™ Neuronal medium (StemCell Technologies).

### Infection of neuronal progenitor cells (NPCs)

Monolayer cultures of NPCs were infected at an MOI of 0.1 with an HSV-1 recombinant virus expressing the reporter genes EGFP and RFP under the control of the HSV-1 promoters ICP0 and gC, respectively (Ref). Two hours after the infection the inocula were removed, cells were washed and cultured in StemDiff Neural progenitor Medium supplemented with the above described alkaloid analogs at the concentration of 10 μM or ACV at 50 μM. Cells were analyzed by flow cytometry at day 3 p.i.

### Infection brain organoids

Fourteen-week old brain organoids were infected singularly in U-bottom low attachment 96-well plates with an HSV-1 DualF construct (6000 pfu/organoid). After 2 h, the inocula were removed, the organoids were washed and cultured in BrainPhys medium supplemented with the tested compounds (10 μM) or ACV (50 μM). The organoids that were infected in the presence of ACV were pretreated with the antiviral for 24 h.

### Organoids viability

Organoids viability was analyzing using calcein AM assay (BioLegend; catalogue # 425201), according to manufacturer’s instructions. Briefly, organoids were exposed singularly in Eppendorf tube containing Calcein-AM 0.01 μM and incubated at 37 °C. After 20 min, organoids were transferred in prewarmed culture medium and incubated for additional 10 min. After incubation, fluorescence signals were recorded using a LEICA DMIL LED Fluorescent microscope through a 0.16NA 4× air objective.

The corrected total cell fluorescence (CTCF) was obtained by measuring EGFP fluorescence in 799-treated and untreated organoids, and normalizing for whole organoid area using equation CTCF = integrated density − (whole organoid area × mean fluorescence of background readings).

### RNA sequencing

NPCs were cultured in StemDiff Neural progenitor Medium, containing compounds **2b**, **8**, (10 µM) or vehicle. All assays were conducted in triplicate. Cells were harvested after 72 h and the cellular RNA was extracted (RNeasy Mini Kits, Qiagen) and quantified using Agilent 4200 TapeStation (Agilent Technologies). Total RNA libraries were generated using the Illumina TruSeq Stranded Total RNA Sample Preparation Guide, Revision E. The first step involved the removal of ribosomal and mitochondrial RNA using biotinylated, target-specific oligos combined with Ribo-Zero rRNA removal beads. Following purification, remaining RNA was fragmented using divalent cations under elevated temperature, which were then copied into first strand cDNA using reverse transcriptase and random primers, followed by second strand cDNA synthesis using DNA Polymerase I and RNase H. Subsequently, a single adenosine base was added to each of the cDNA fragments, followed by ligation of an adapter. The products were purified and enriched with PCR to create the final cDNA library. The cDNA libraries were validated using KAPA Biosystems primer premix kit with Illumina-compatible DNA primers and Qubit 2.0 fluorimeter. Quality was examined using an Agilent Bioanalyzer Tapestation 2200. The cDNA libraries were pooled at a final concentration 1.8 pM. Cluster generation and 100 bp paired-read dual-indexed sequencing was performed on Illumina NextSeq 500 (Children’s Hospital of Pittsburgh, University of Pittsburgh).

Sequencing read quality was assessed using fastQC v0.11.4 and CLCbio v11.0.1 software. The average number of reads per sample was 39.5 million (SD = 4.8 million reads). Sequences were trimmed based on quality score using the modified-Mott trimming algorithm as implemented in CLC bio software, using a trim cutoff error probability of 0.05. Ambiguous bases were trimmed using a post trim maximal ambiguous base cutoff of 2. The trimmed reads were then mapped to the human genome GRCh38/hg38, using sequence and annotation provided by Ensembl (release 82). Approximately 92% of reads were mapped in pairs (SD = 1.14) across all samples, and 97.7% of reads were mapped in total (SD = 0.45). The data were deposited in NCBI’s Gene Expression Omnibus database (GSE201156).

### Quantitative RT-PCR (qRT-PCR)

Quantitative reverse transcription PCR (qRT-PCR) was used to determine gene expression analysis using Taqman Assays (Thermo Fisher, US) specific for TP53 (ID Hs01034249_m1), SIRT1 (Hs01009006_m1) and BCL2 (Hs00608023_m1) genes and ATF4 with primers set: FWD 5′TCAAACCTCATGGGTTCTCC3′ and REV: 5′GTGTCATCCAACGTGGTCAG3′. Every sample had 3 or 4 biological replicates. Reverse transcription reaction was obtained from 100 ng of total RNA using random hexamers and SuperScript IV (Thermo Fisher). The conditions for the RT reaction were priming at 65 °C for 5 min, following 55 °C for 10 min and 80 °C for 10 min; then held on 4 °C. 2 μL of diluted cDNA (1:3) was added to Luna Universal Probe qPCR Master Mix or Luna Universal qPCR Master Mix (NBE, USA) to the total volume of 10uL. PCR was performed in CFX96 (BioRad, USA) under following conditions: 95 °C for 60 s, followed by 40 cycles of 95 °C for 15 s and 30 °C for 1 min. The levels of gene expression were determined using Ct (threshold cycle). The ∆Ct was calculated by subtracting the Ct of GAPDH (FWD: 5′ACCCACTCCTCCACCTTTG3′, REV: 5′CTCTTGTGCTCTTGCTGG3′) from the Ct of interest gene. ∆∆C was calculated by subtracting ∆C of the reference sample from the ∆C of the control samples. Fold change was presented by the following equation 2^−ΔΔ^ Ct.

### Assessment of antiviral activity in a mouse ocular model of HSV-1 infection

ND4 Swiss mice, 4–6 weeks of age, (Envigo labs) were infected by the ocular route. Mice were anesthetized with isoflurane and the cornea were lightly scarified with a 23 ga. needle. 1 × 10^5^ pfu (in 5 µl) of HSV-1 strain *17syn*+ was applied to the corneal surface. At the same time, one group of mice (n = 10) were treated with 2 µL of a 50 µM solution of compound **8** in DMSO and the other group (n = 10) were treated with 2 µL DMSO (vehicle), applied to the eyes. The eyes were treated with either vehicle or compound **8** each day for 4 days. In order to assess the effect of compound **8** on the course of the infection, the eyes were swabbed daily with a Dacron swab, and the virus eluted in MEM medium. The swab eluate was assessed for the presence of infectious virus by standard plaque assay on rabbit skin cell monolayers.

### Statistical analysis

All statistical analysis were performed in GraphPad Prism 9. One-way analysis of variance (ANOVA) followed by post hoc analysis for analysis shown in Figs. 1, 3, and 5 (see figures for details).

## Supplementary Information

Supplementary Information.

## Data Availability

The datasets used and/or analysed during the current study available from the corresponding author on reasonable request.
