# Supplementary material for: Truncated ring-A amaryllidaceae alkaloid modulates the host cell integrated stress response, exhibiting antiviral activity to HSV-1 and SARSCoV-2
Source: Sci Rep. 2023 Jan 30;13:1639. doi: 10.1038/s41598-023-28691-0 (PMC9885069; doi:10.1038/s41598-023-28691-0)
Supplement: Supplementary file 1 — Supplementary Information. [file 41598_2023_28691_MOESM1_ESM.docx]

**Supporting Information**

Truncated ring-A Amaryllidaceae alkaloid modulates the host cell integrated stress response, exhibiting antiviral activity to HSV-1 and SARSCoV-2

James McNulty, Chanti Babu-Dokuburra, Jon Scattolon, Carlos Zepeda-Velazquez, Maribeth A. Wesesky, Jill K. Caldwell, Wenxiao Zheng, Jadranka Milosevic, Paul R. Kinchington, David C. Bloom, Vishwajit L. Nimgaonkar, Leonardo D’Aiuto

**A) Chemical synthesis and analysis.**

**Solvents and reagents:** All chemicals and solvents were purchased from Acros, Aldrich, J.T. Baker, Caldeon, Solvay and Fluka and used as received with the following exceptions: deuterated solvents were obtained from ACP Chemicals, Toronto, Canada. Tetrahydrofuran (THF), diethyl ether (Et_2_O), and toluene were distilled from sodium/benzophenone under an atmosphere of dry nitrogen; dichloromethane (CH_2_Cl_2_) was distilled from calcium hydride under an atmosphere of dry nitrogen; methanol (MeOH) was distilled from magnesium turnings under an atmosphere of dry nitrogen; triethylamine (NEt_3_), *N*,*N*-diisopropylethylamine (Hünig’s base) and pyridine were distilled from potassium hydroxide under an atmosphere of dry nitrogen; solid sodium hydride (NaH) was obtained by filtration and washing with *n*-hexanes.

**Reaction handling:** All non-aqueous reactions were performed in flame dried round bottom flasks or in non-flame-dried amber 1.5-dram vials. Reactions were magnetically stirred and monitored by thin-layer chromatography (TLC) unless otherwise noted. TLC was performed on Macherey-Nagel silica gel 60 F_254_ TLC aluminum plates and visualized with UV fluorescence quenching and potassium permanganate (KMnO_4_) or 2,4-dinitrophenylhydrazine or *p*-anisaldehyde stains.^1^ Concentrations under reduced pressure were performed by rotary evaporation at 40 °C at the appropriate pressure unless otherwise noted. Column chromatographic purification was performed as flash column chromatography with 0.3–0.5 bar pressure using Silicycle silica gel (40–63, 60 Å) or EcoChrom silica gel (12–26, 60 Å). Distilled technical grade solvents were employed. The yields given refer to chromatographically purified and spectroscopically pure compounds unless stated otherwise.

**Nuclear Magnetic Resonance (NMR) spectroscopy:** ^1^H, ^13^C{1H}, DEPTq, COSY, HSQC, and HMBC NMR spectra were obtained on Bruker DRX-500, AV-600, and AV-700 spectrometers. All ^1^H NMR spectra were referenced relative to SiMe_4_ through a resonance of the employed deuterated solvent or impurity of the solvent; chloroform (7.26 ppm), DMSO (3.33 ppm) and methanol (3.31 ppm) for ^1^H NMR; chloroform (77.00 ppm), DMSO (39.52 ppm) and methanol (49.00 ppm) for ^13^C NMR. All NMR spectra were obtained at RT (*ca*. 22 °C) unless otherwise specified. The data is reported as (s = singlet, d = doublet, t = triplet, m = multiplet or unresolved, br = broad signal, coupling constant(s) in Hz, integration). ^13^C-NMR spectra were recorded with complete ^1^H-decoupling. Service measurements were performed by the NMR service team of the Nuclear Magnetic Resonance Facility at McMaster University by Dr. Bob Berno and Dr. Hilary A. Jenkins.

**Mass spectrometry:** Mass spectrometric analyses were performed as high-resolution ESI measurements on a Waters/Micromass QTof Global Ultima (quadrupole time-of-flight mass spectrometer) or high-resolution EI in a Waters/Micromass GCT (time-of-flight mass spectrometer) instrument by the mass spectrometry service of the McMaster Regional Centre for Mass Spectrometry (MRCMS) at McMaster University by Megan Fair and Leah Allan under the supervision of Dr. Kirk Green.

**Enantiomeric ratios:** Enantiomeric ratios were determined using an Agilent 1220 Infinity HPLC manual injection with a variable wavelength detector, using a Daicel Chiralpak® AD-H column (150 x 4.6 mm, 5µ), n-hexane/iPrOH (80:20) as a mobile phase; flow rate 0.75 ml/min, column temperature 25°C, λ236 nm, sample 1 mg / 1ml dissolved in the mobile phase.

**Optical rotations:** Optical rotations were measured on a Perkin-Elmer 241 MC polarimeter, [*α*] is given in degcm^3^g^−1^dm^−1^ and *c* is given in gcm^−3^.

Synthetic Procedures:

**Azidopropan-2-one (11).**

Sodium azide (2.485 g, 38.23 mmol) was added to a solution of 1-bromopropan-2-one (2.54 mL, 31.63 mmol) in acetone (10 mL) and water (20 mL). The reaction mixture was stirred at RT for 24 h and acetone was removed using nitrogen stream. The remaining aqueous solution was extracted with CH_2_Cl_2_ (2 x 25 mL). The combined organic extracts were washed with brine, dried over anhydrous Na_2_SO_4_, and filtered. The filtrate had a nitrogen stream blown over it until all solvent was removed, yielding (2.505 g, 80% yield) the title compound as a colorless liquid. This compound is known and matches the reported spectroscopic data.^1^

**Tripropyl-(2,2-diethoxyethyl)-phosphonium bromide**

 A solution of bromoacetaldehyde diethyl acetal (9.60 g, 48.7 mmol) and tri-*n*-propylphosphine (8.58 g, 53.6 mmol) in THF (27 mL) was heated for 24 h at 60 °C. After cooling, the solvent and the residual tri-*n*-propylphosphine were removed on the rotary evaporator (75 °C at 5 mbar). The obtained yellow oil was dried *in vacuo* (0.1 mbar) isolating 16.38 g of phosphonium bromide as a white solid. No further purification was necessary. Storage temperature: 0 °C. This compound is known and matches the reported spectroscopic data.

**(*E*)-3-Methoxyphenyl-cinnamaldehyde (10)**

3-Methoxybenzaldehyde (1.822 g, 13.40 mmol) and tributyl-(2,2-diethoxyethyl)-phosphonium bromide (5.99 g, 16.7 mmol) were dissolved in anhydrous THF (20 mL). Sodium hydride (0.97 g, 40.3 mmol) was added to the reaction mixture over a period of 10 min, maintaining the temperature below 30 °C, and the suspension was stirred for 24 h at RT. Water (25 mL) was added to the mixture, and the mixture was extracted with CH_2_Cl_2_ (3 × 25 mL). The extracts were combined and washed again with water (2 x 25 mL). The organic layers were dried using Na_2_SO_4_, filtered and concentrated under vacuum to afford diethyl acetal of aldehyde 3.19 g of red oil. 20 mL of 2 M HCl was added and stirred for 1 h at RT. When the reaction was completed, the reaction mixture was extracted with CH_2_Cl_2_ (3 × 25 mL). compound was separated by column chromatography to afford 3-methoxy-cinnamaldehyde **10** (2.063 g, 95% Yield). Data were in accord with reported spectroscopic data,^2^ ^1^H NMR (600 MHz, CDCl_3_) δ 9.67 (d, *J* = 7.7 Hz, 1H, *H*C*=*O), 7.41 (d, *J* = 15.9 Hz, 1H, C*H*=CH-CHO), 7.31 (t, *J* = 7.9 Hz, 1H, Ar-H)), 7.13 (d, *J* = 7.6 Hz, 1H,Ar-H ), 7.05 (s, 1H, Ar-*H*), 6.96 (dd, *J* = 8.2, 1.8 Hz, 1H, CH=*CH*-CHO 6.67 (dd, *J* = 15.9, 7.7 Hz, 1H, Ar-*H*), 3.81 (s, 3H, -OC*H*_3_). HRMS (ESI): exact mass calculated for C_10_H_10_O_2_Na [(M + Na)+], 185.0578; found 185.0566.

**(2*R*,3*R*,5*S*)-2-Azido-5-hydroxy-3-(3-methoxyphenyl)cyclohexanone (9)**

**A solution of 3-methoxy-cinnamaldehyde **12** (0.758 g, 4.68 mmol) and (*R*)-(+)-α,α-diphenyl-2-pyrrolidinemethanol trimethylsilyl ether (0.145 g, 0.45 mmol) in CH_2_Cl_2_ (6.2 mL) was stirred for 10 min, after which it was cooled to -20 °C. Azidopropan-2-one **11** (0.442 g, 4.46 mmol) was added dropwise over 5 min. The brown solution was stirred for 20 min at RT and quinidine (0.145 g, 0.45 mmol) was added in one portion. The reaction was stirred at RT for 24 h, after which TLC (CH_2_Cl_2_ /MeOH 98:2) showed full conversion. The CH_2_Cl_2_ was carefully evaporated (30 °C, 32 mbar) yielding a brown oil that was purified by flash chromatography (eluent CH_2_Cl_2_ /MeOH 100:0 to 95:5,) to afford product **9** (0.671g, 55% yield). [α]^23^_D_ = +87 (c = 0.82, MeOH, l = 1 dm). ^1^H NMR (600 MHz, CDCl_3_) δ 7.30 (t, *J* = 7.9 Hz, 1H, Ar-*H*,), 6.89 (d, *J* = 7.6 Hz, 1H, Ar-*H*), 6.86 – 6.84 (m, 1H, Ar-*H*), 6.83 (s, 1H, Ar-*H*), 4.58 (m, 1H, C*H*-OH ), 4.10 (d, *J* = 12.0 Hz, 1H, C*H*-N_3_), 3.82 (s, 3H, -OC*H*_3_), 3.46 (td, *J* = 12.1, 4.3 Hz, 1H, Ph-C*H*), 2.83-2.69 (m, 2H, C*H*_2_CO), 2.27 – 2.13 (m, 2H, Ph-CH-C*H*_2_), 1.72 (s, 1H,-O*H*). ^13^C NMR (151 MHz, CDCl_3_) *δ* 202.92, 159.99, 142.09, 130.02, 119.52, 113.59, 112.61, 71.21, 67.88, 55.27, 48.14, 44.70, 39.33. HRMS (ESI): exact mass calculated for C_13_H_15_N_3_O_3_Na [(M + Na)^+^], 284.1011; found 284.1006.

**(2*S,*3*S*,5*R*)-2-Azido-5-hydroxy-3-(3-methoxyphenyl)cyclohexanone (*ent-9*)**

The title compound was synthesized following the above procedure but employing (*S*)-(+)-α,α-diphenyl-2-pyrrolidinemethanol trimethylsilyl ether as catalyst. [α]^23^_D_ = -82 (c = 1, MeOH, l = 1 dm).

**Methyl ((1*R*,2*R*,4*S*)-4-hydroxy-2-(3-methoxyphenyl)-6-oxocyclohexyl)carbamate (13)**

In a 50 ml RBF, azide **9** (0.391 g, 1.5 mmol), dimethyl dicarbonate (0.600 g, 4.5 mmol) and 10 % Pd/C (0.12 g, 0.10 mmol) were suspended in methanol (15 mL). The vessel was sealed and subjected to hydrogen balloon (1 atm) with vigorous stirring for 12 h, after which TLC (CH_2_Cl_2_ /MeOH 95:5) showed full conversion. The suspension was filtered through a celite® pad and carefully evaporated (20 °C, 0.1 mbar). The translucent grey oil was purified by flash chromatography (eluent CH_2_Cl_2_ /MeOH 100:0 to 97:3) giving the product as a translucent oil carbamate **13** (0.325 g, 75% yield) [α]^23^_D_ = -23 (c = 2, MeOH, l = 1 dm). e.e.: τmajor = 7.49 min, τminor = 12.3 min (>99.5% e.e.), AD-H column (150 x 4.6 mm, 5µ), n-hexane/iPrOH (80:20) as a mobile phase; flow rate 0.75 ml/min, column temperature 25°C, λ236 nm, sample 1 mg / 1ml dissolved in the mobile phase. ^1^H NMR (600 MHz, CDCl_3_) δ 7.24 (t, *J* = 7.9 Hz, 1H, Ar-*H*), 6.86 (d, *J* = 7.5 Hz, 1H, Ar-*H*), 6.80 (d, *J* = 12.7 Hz, 1H, Ar-*H*), 6.78 (dd, *J* = 8.2, 2.3 Hz, 1H, Ar-*H*), 5.25 (d, *J* = 8.7 Hz, 1H), 4.61 (t, *J* = 9.2 Hz, 1H), 4.53 (m, 1H), 3.78 (s, 3H, Ar-OC*H*_3_), 3.48 (s, 3H, NHCOOC*H*_3_ ), 3.28 (dd, *J* = 19.7, 8.5 Hz, 1H, Ph-C*H*), 3.04-2.95 (bs, 1H, O*H*), 2.83 (d, *J* = 13.1 Hz, 1H, COC*H*_2_), 2.70 (d, *J* = 13.9 Hz, 1H, COC*H*_2_), 2.19 (t, *J* = 11.2 Hz, 2H, Ph-CH-C*H*_2_). ^13^C NMR (151 MHz, CDCl_3_) δ 206.01, 159.64, 157.02, 142.30, 129.62, 119.76, 113.56, 112.33, 68.17, 63.17, 55.16, 52.16, 48.19, 45.44, 40.29. HRMS (ESI): exact mass calculated for C_15_H_19_NO_5_Na [(M + Na)^+^], 316.1161; found 316.1158.

**(2*S*,4*R*,4*aR*,10*aR*)-4,9-Bis(3-methoxyphenyl)-1,2,3,4,4*a*,5,10,10*a*-octahydrophenazin-2-ol (18)**

By-product **18** (0.062g, 10% yield). [α]^23^_D_ = +97 (c = 1, MeOH, l = 1 dm). ^1^H NMR (600 MHz, CDCl_3_) δ 7.40 (t, *J* = 7.9 Hz, 1H, Ar-*H*), 7.31 (t, *J* = 7.8 Hz, 1H, Ar-*H*), 7.03 (d, *J* = 7.5 Hz, 1H, Ar-*H*), 7.00 (s, 1H, Ar-*H*), 6.95 (d, *J* = 8.3 Hz, 1H, Ar-*H*), 6.90 (d, *J* = 7.5 Hz, 1H, Ar-*H*), 6.86 (d, *J* = 8.9 Hz, 1H, Ar-*H*), 6.84 (s, 1H, Ar-*H,*), 6.75 (t, *J* = 7.7 Hz, 1H, Ar-*H*), 6.61 (d, *J* = 7.5 Hz, 1H, Ar-*H*), 6.40 (d, *J* = 7.9 Hz, 1H, Ar-*H*), 4.49-4.29 (bs, 1H, N*H*) 4.15-4.11 (m, 1H, Ph-C*H* ), 3.41 (td, *J* = 3.2, 12.0 Hz, 1H, Ph-CH-CH(NH)-C*H*-NH), 2.21 (d, *J* = 14.0 Hz, 1H, Ph-CH-C*H*_2_), 2.13 (d, *J* = 15.0 Hz, 1H, CH(NH)-C*H*_2_), 1.96-1.87 (m, 2H, CH(NH)-C*H*_2_, Ph-CH-C*H*_2_), 1.70 (s, 1H, CH-O*H*). ^13^C NMR (151 MHz, CDCl_3_) δ 159.96 (2C), 144.20, 140.16, 132.64, 130.04, 129.80, 129.20, 127.83, 121.33, 120.60, 120.37, 119.57, 114.03, 113.93, 113.91(2C), 113.65, 112.34, 67.03, 56.14, 55.28 (2C), 49.52, 39.43, 39.26, 36.68. HRMS (ESI): exact mass calculated for C_26_H_28_NO_3_Na [(M + Na)^+^], 439.1988; found 439.1978.

Note: When we used 0.15 equiv of 10 mol% Pd/C and reaction was stirred to 32 h, we have not observed by-product **18**.

**Methyl ((1*S*,2*S*,4*R*)-4-hydroxy-2-(3-methoxyphenyl)-6-oxocyclohexyl) carbamate (*ent-13*)**

The title compound was made from ***ent*- 9.** [α]^23^_D_ = +21 (c = 2, MeOH, l = 1 dm). Enantiomeric ratio was determined using chiral HPLC: minor = 7.59 min, major = 12.3 min (>99.5% e.e.), AD-H column (150 x 4.6 mm, 5µ), n-hexane/iPrOH (80:20) as a mobile phase; flow rate 0.75 ml/min, column temperature 25°C, λ236 nm, sample 1 mg / 1ml dissolved in the mobile phase.

**Methyl ((1*R*,2*R*,3*R*)-3-hydroxy-3'-methoxy-1,2,3,6-tetrahydro-[1,1'-biphenyl]-2-yl)carbamate (14)**

In a 50 mL RBF, the carbamate **13** (0.360 g, 1.23 mmol) was dissolved in CH_2_Cl_2_ (18 mL) under nitrogen and cooled in an ice bath. Methanesulfonyl chloride (0.123 mL, 1.59 mmol) was added in one portion, and then Hünig’s base (0.642 mL, 3.69 mmol) was added dropwise. The resulting solution was stirred for 10 h at RT, then poured into water (10 mL) and the layers separated. The organic phase was washed with 1 M HCl (1 mL) then brine (10 mL) and dried over Na_2_SO_4_. Concentration under reduced pressure gave the crude product as a translucent oil (0.305g, 95% yield). This product was used with out further purification. In a 50 mL RBF, ketone (0.305 g, 1.10 mmol) was dissolved in THF 15 ml, and cooled to 0 ^o^C and added Lithium tri-tert-butoxyaluminum hydride (0.846 g, 3.30 mmol). After 12h of stirring, saturated aqueous NH_4_Cl was added to quench the reaction. The product was extracted with EtOAc (3 x 15 mL) and the combined organic layer was washed with brine (10 mL) and dried over Na_2_SO_4_. The solvent was removed under reduced pressure and the residue was purified by column chromatography (CH_2_Cl_2_ / MeOH; 100:0 to 97:3) to afford the product **14** (0.291 g, 95% yield) as a translucent oil. [α]^23^_D_ = -104.3 (c = 1, MeOH, l = 1 dm). ^1^H NMR (600 MHz, CDCl_3_) δ 7.24 (t, *J* = 6.9 Hz, 1H, *Ar-H*), 6.79 (m, 2H, Ar-*H* ), 6. 6.76 (dt, *J* = 3.9, 1.8 Hz, 1H, Ar*-H*), 5.82- 5.75 (m, 1H, CH2-C*H*=), 5.72 (dt, *J* = 3.7, 2.1 Hz, 1H, =C*H*-CH-OH), 4.59 (brs, 1H, NH), 4.33 (br s, 1H, C*H*-OH), 4.25 (brs, -O*H*) 3.85 (ddd, 1H, , J = 13.4, 7.8, 6.5 Hz, 1H, C*H*-NH), 3.80(s, 3H, Ar-OC*H*_3_), 3.55 (s, 3H, HNCOO-C*H*_3_), 2.86 (td, J = 10.3, 6.5 Hz, 1H, Ar-C*H*), 2.42 -2.29 (m, 2H, Ar-CH-C*H*_2_). ^13^C NMR (151 MHz, CDCl_3_) δ 160.03, 158.54, 142.65, 130.05, 129.40, 126.92, 120.00, 113.51, 112.57, 74.09, 58.60, 55.22, 52.44, 44.82, 34.96. HRMS (ESI): exact mass calculated for C_15_H_19_NO_4_Na [(M + Na)^+^], 300.1212; found 300.1208.

**(1*S*,2*R*,3*S*,4*R*,5*R*)-4-((Methoxycarbonyl)amino)-5-(3-methoxyphenyl)cyclohexane-1,2,3-triyl triacetate (15).**

In a 50 mL RBF, vinyl alcohol **14** (0.260 g, 0.94 mmol) was dissolved in CH_2_Cl_2_ (20 mL) along with NaHCO_3_ (0.158 g, 1.88 mmol). To the stirred suspension was added *m*-CPBA (0.422g, 1.88 mmol) at RT. The resulting suspension was stirred vigorously for 24 h. A 20% (w/v) aqueous solution of sodium sulfite (10 mL) was added, and the resulting two-phase mixture was stirred vigorously for 15 min. The two layers were separated, and the aqueous layer was extracted with CH_2_Cl_2_ (2 x 10 mL). The combined organic layers were washed with a 20% (w/v) aqueous solution of sodium sulfite (10 mL) and a 5% (w/v) aqueous solution of NaHCO_3_ (2 x 10 mL), dried with anhydrous Na_2_SO_4_ and evaporated under reduced pressure (20 °C, 32 mbar) to give a 4:1 diastereomeric mixture (0.234 g) of compounds by TLC and which was used for next step without further purification.

To a solution of epoxide mixtures (0.234 g, 0.80 mmol) in 5.1 mL of water was added sodium benzoate (0.009 g, 0.06 mmol). The mixture was heated at 90-95 °C for 16 h. TLC showed full conversion. The solution was cooled to RT, the water was removed in vacuo, and the light pink residue which single trihydroxy compound (0.

To a solution of trihydroxy compound (0.140 g, 0.80 mmol) in pyridine (0. 386 mL, 4.80 mmol) was added acetic anhydride (0.243 mL, 4.80 mmol). The reaction mixture was stirred at RT for 16 h. After that the pyridine was removed in vacuo (0.1 mbar). The residue was dissolved in EtOAc (20 mL) and washed with saturated NaHCO_3_ (2 x 10 mL) and water (10 mL). The solvent was removed in vacuo and the product purified by flash column chromatography (CH_2_Cl_2_ / MeOH; 100:0 to 98:2) to afford triacetate **15** (0.316 g, over 3 steps **77** % yield). [α]^23^_D_ = -12 (c = 1, MeOH, l = 1 dm).

^1^H NMR (600 MHz, CDCl_3_) δ 7.22 (t, *J* = 8.0 Hz, 1H, Ar-*H*), 6.84 (t, *J* = 8.0 Hz, 1H, Ar-*H*), 6.80-6.75 (m, 2H, Ar-*H*), 5.37 (t, *J* = 2.9 Hz, 1H, C*H*-OAc), 5.23 (d, *J* = 9.2 Hz, 1H, C*H*-OAc), 5.15 – 4.96 (m, 1H, C*H*-OAc), 4.44 (d, *J* = 33.5 Hz, 1H, N*H*), 4.25 (m, 1H, C*H*NH), 3.80 (s, 3H Ar-OC*H*_3_), 3. 46 (s, 3H, O=COC*H*_3_), 2.95 (m, 1H. Ar-C*H*), 2.38 (s, 3H, O=C-C*H*_3_), 2.17 (s, 3H, O=C-C*H*_3_), 2.17-2.12 (m, 1H, ), 2.03-2.02(m, 1H, Ar-CH-C*H*_2_), 2.01 (s, 3H, O=C-C*H*_3_). HRMS (ESI): exact mass calculated for C_21_H_27_NO_9_Na [(M + Na)^+^], 460.1584; found 460.1578.

**(2*S*,3*R*,4*S*,4*aR*,10*bR*)-9-Methoxy-6-oxo-1,2,3,4,4*a*,5,6,10*b*-octahydrophenanthridine-2,3,4-triyl triacetate (16)**

Into a 50 mL RBF, compound **15** (0.140 g, 0.32 mmol) and DMAP (0.117 g, 0.96 mmol) were dissolved in CH_2_Cl_2_ (8 mL) at 0 °C. A 1.0 M solution of Tf_2_O in CH_2_Cl_2_ (1.597 mL, 1.60 mmol) was added dropwise to the reaction mixture over a period of 10 min. The reaction was stirred for 16 h at RT. The solvent was evaporated, and the residue treated with a mixture of THF (5 mL) and 1 M HCl (1 mL). After stirring for 1 h at RT, the mixture was partitioned between a saturated aqueous solution of NaHCO_3_ (1 mL) and CH_2_Cl_2_ (20 mL). The organic phases were combined, dried with anhydrous Na_2_SO_4_ and concentrated. The Regio isomers (compounds **16** and **17**) were purified by flash column chromatography (CH_2_Cl_2_ / MeOH; 100:0 to 98:2).  Compound **16** (major), 0.066 g, 51% yield; Rf=0.35 (Hexane/ethyl acetate, 1:1); [α]^23^_D_ = +34 (c = 0.1, MeOH, l = 1 dm).^1^H NMR (600 MHz, CDCl_3_)**δ**8.096 (d, J = 8.65 Hz, 1H), 6.939 (dd, J = 8.54, 2.09 HZ, 1H), 6.778 (m, 1H), 6.582 (s, 1H), 5.513 (t, J = 2.94 HZ, 1H), 5.254 (m, 2H), 3.884 (s, 3H), 3.855 (dd, J = 11.1, 12.4 HZ, 1H), 3.287 (d, J = 3.74, 12.23 HZ, 1H), 2.561 (dt, J = 3.3, 14.6 HZ, 1 H), 2.70 (s, 3H), 2.126 (s, 3H), 2.088 (s, 3H), 2.008 (m, 1H); ^13^C NMR (151 MHz, CDCl3) **δ**170.27, 169.46, 169.20, 166.13, 163.35, 141.90, 130.75, 121.32, 112.07, 109.67, 77.23, 77.02, 76.81, 71.76, 68.67, 67.51, 55.53, 52.69, 35.02, 26.45, 21.08, 20.85, 20.72. HRMS (ESI): exact mass calculated for C_20_H_23_NO_8_Na [(M + Na)+], 428.1321; found 428.1316.

**(2*S*,3*R*,4*S*,4*aR*,10*bR*)-7-Methoxy-6-oxo-1,2,3,4,4*a*,5,6,10*b*-octahydrophenanthridine-2,3,4-triyl triacetate (17)**

 Compound **17** (minor), 0.019 g, 15% yield, Rf**=**0.30 (Hexane/ethyl acetate 1:1), [α]^23^_D_ = +41 (c = 1, MeOH, l = 1 dm). ^1^H NMR (600 MHz, CDCl_3_) δ 7.50 (m, 1H), 7.00(d, J = 8.57 HZ, 1H), 6.869 (d, J = 7.76 HZ, 1H), 6.386 (s, 1H), 5.441 (t, 3H), 5.219 (m, 2H), 3.957 (s, 3H), 3.746 (t, J = 11.35 HZ, 1H), 3.223 (td, J = 3.98, 12.6 HZ, 1H), 2.545 (dt, J = 3.4, 14.3 HZ, 1H), 2.163 (s, 3H), 2.08 (s, 3H), 2.06 (s, 3H), 1.962 (m, 1H). ^13^C NMR (151 MHz, CDCl_3_) δ 170.39, 169.46, 169.19, 164.37, 160.35, 142.56, 133.39, 117.27, 115.42, 111.50, 71.66, 68.66, 67.46, 56.28, 52.01, 35.87, 26.79, 21.08, 20.78, 20.72. HRMS (ESI): exact mass calculated for C_20_H_23_NO_8_Na [(M + Na)^+^], 428.1321; found 428.1314.

**(2*S*,3*R*,4*S*,4*aR*,10*bR*)-2,3,4-Trihydroxy-9-methoxy-1,3,4,4*a*,5,10*b*-hexahydrophenanthridin-6(2H)-one (5)**

Compound **16** (0.005 g (0.012 mmol) and potassium carbonate (0.002 g, 0.01 mmol) were dissolved in MeOH (1 mL) and stirred at RT. TLC analysis showed full conversion after 6 h. The mixture was concentrated under a flow of N_2_. The white product was dissolved in 9:1 CH2Cl2 / MeOH and filtered through a pad of silica to afford 9-metoxy trihydroxy compound **5** (0.0032 g, 96% yield). [α]^23^_D_ = +52 (c = 0.25, MeOH, l = 1 dm). ^1^H NMR (700 MHz, DMSO) δ 7.81 (d, *J* =8.5 Hz, 1H), 6.90 (d, *J* = 8.5, Hz, 1H), 6.87 (s, 1H), 6.82 (s, 1H), 5.14 (bm, 3H), 3.91 (d, *J* = 2.4 Hz, 1H), 3.82 (s, 3H), 3.76-3.71 (m, 2H), 2.95 (dd, *J* = 11.9, 9.3 Hz, 1H), 2.18 (dt, *J* = 13.0, 3.2 Hz, 1H), 1.70 (td, *J* = 13.0, 2.2 Hz, 1H). ^13^C NMR (176 MHz, DMSO) δ 164.60, 162.35, 144.41, 129.45, 122.07, 111.69, 109.23, 71.72, 69.89, 68.66, 55.37, 55.07, 40.06, 34.53, 28.03. HRMS (ESI): exact mass calculated for C_14_H_17_NO_5_Na [(M + Na)^+^], 302.1004; found 302.0986.

**(2*S*,3*R*,4*S*,4*aR*,10*bR*)-2,3,4-Trihydroxy-7-methoxy-1,3,4,4*a*,5,10*b*-hexahydrophenanthridin-6(2H)-one (7)**

 Compound **17** (0.005 g (0.012 mmol) and potassium carbonate (0.002 g, 0.01 mmol) were dissolved in MeOH (1 mL) and stirred at RT. TLC analysis showed full conversion after 6 h. The mixture was concentrated under a flow of N_2_. The white product was dissolved in 9:1 CH_2_Cl_2_ / MeOH and filtered through a pad of silica 7-methoxy trihydroxy compound **7** (0.0032 g, 96% yield). [α]^23^_D_ = +56 (c = 0.25, DMSO, l = 1 dm). ^1^H NMR (600 MHz, DMSO) δ 7.47 (s, 1H, CON*H*), 7.45 (t, *J* = 8.0 Hz, 2H, Ar-*H*), 7.02 (d, *J* = 8.5 Hz, 1H, Ar-*H*) 6.91 (d, *J* = 7.8 Hz, 1H, Ar-*H*), 3.91 (dd, *J* = 3.2, 6,25 Hz, 1H, CH_2_C*H*-OH), 3.79 (s, 3H, Ar-OMe), 3.77 -3.75 (m, 1H, CH2CHOHC*H*OH), 3.25 -3.19 (dd, *J* = 10.2, 3.0 Hz, 1H, CHNHC*H*OH) 2.85 (td, *J* = 12.3, 3.8 Hz, 1H, ), 2.11 (dd, *J* = 10.0, 3.2 Hz, 1H), 1.67 (td J= 3.2 13.7, 1H). ^13^C NMR (151 MHz, DMSO) δ 163.04, 159.12, 145.01, 132.35, 118.24, 115.49, 111.17, 71.70, 69.40, 68.63, 55.70, 54.73, 35.55, 28.55. HRMS (ESI): exact mass calculated for C_14_H_17_NO_5_Na [(M+ Na)^+^], 302.1004; found 302.0988.

**(2*S*,3*R*,4*S*,4*aR*,10*bR*)-9-Hydroxy-6-oxo-1,2,3,4,4*a*,5,6,10*b*-octahydrophenanthridine-2,3,4-triyl triacetate (19)**

Compound**** **16** (0.050 g, 0.123 mmol) was dissolved in 1:1 ratio of dry CH_2_Cl_2_ and dry Benzene. After addition of Aluminium chloride (0.049 g, 0.369 mmol) under argon at 0 ^o^C and n-Bu4N^+^I^-^ (0.2 7g, 0.738 mmol) was added under argon was added to the reaction mixture over a period of 10 min at 0 ^o^C. The reaction mixture turned a red colour and was stirred for 3 hours at RT. After confirming full conversion of starting material with TLC, the reaction mixture was quenched with water, and stirred for 2 N HCl for 0.5 h, and was extracted with EtOAc (3x 10 mL). The combined organic phase was washed with brine, dried over Na_2_SO_4_, filtered through Dowex® 50WX8 hydrogen form and concentrated in vacuo and purified by flash column chromatography (CH_2_Cl_2_ / MeOH; 100:0 to 98:2) to afford phenolic compound **19** (0.042g, 88% Yield). [α]^23^_D_ = +45 (c = 0.9, MeOH, l = 1 dm). ^1^H NMR (700 MHz, CDCl_3_) δ 7.99 (t, *J* = 7.8 Hz, 1H, ArH), 6.86 (dd, *J* = 8.4, 1.9 Hz, 1H, ArH), 6.85 -6.77 (bs, 1H, CON*H*), 6.75 (s, 1H, Ar*H*), 5.46 (t, *J* = 3.1 Hz, 1H, C*H*-OAc), 5.23 (dd, *J* = 10.8, 2.9 Hz, 1H, C*H*-OAc), 5.21 (dd, *J* = 5.9, 2.8 Hz, 1H, C*H*-OAc), 3.85 (dd, *J* = 12.5, 11.2 Hz, 1H C*H*-N), 3.67 (dd, *J* = 25.2, 9.1 Hz, 1H, Ph-O*H*), 3.24 (td, *J* = 12.7, 3.5 Hz, 1H, Ar-C*H*), 2.50 (dt, *J* = 14.4, 3.0 Hz, 1H, C*H*_2_), 2.15 (s, 3H, COC*H*_3_), 2.11 (s, 3H, COC*H*_3_), 2.08 (m, 3H, COC*H*_3_) 1.98-1.93 (m, 1H, C*H*_2_). ^13^C NMR (176 MHz, CDCl_3_) δ 170.27, 169.46, 169.17, 166.72, 161.01, 142.60, 131.19, 114.66, 114.66, 110.94, 71.48, 68.53, 67.46, 52.78, 34.69, 26.40, 21.07, 20.84, 20.71. HRMS (ESI): exact mass calculated for C_19_H_21_NO_8_Na [(M + Na)^+^], 414.1165; found 414.1156.

**(2*S*,3*R*,4*S*,4*aR*,10*bR*)-2,3,4,9-Tetrahydroxy-1,3,4,4*a*,5,10*b*-hexahydrophenanthridin-6(2H)-one (6)**

**** Compound **19** (0.0047 g (0.012 mmol) and potassium carbonate (0.002 g, 0.010 mmol) in MeOH (1 mL) was stirred at RT until a white solid precipitated. TLC analysis showed full conversion after 6 h. The mixture was concentrated under a flow of N_2_. The white product was dissolved in 9:1 CH_2_Cl_2_ / MeOH and filtered through a pad of silica. The white solid was recrystallized from methanol to afford trihydroxy compound **6** (0.003 g, 94% yield). [α]^23^_D_ = + 31 (c = 0.25, MeOH, l = 1 dm). ^1^H NMR (700 MHz, DMSO) δ 7.75 (d, *J* = 8.54 Hz, 1H), 6.75(bs, 1H), 6.74 (dd, *J* = 8.54, 2.3 Hz, 1H), 6.71 (s, 1H), 3.94 (dd, *J* = 3.1, 5.6 Hz, 1H), 3.77-3.74 (m, 2H), 3.36 (dd, *J* = 12.4, 9.4 Hz, 1H), 2.94 (td, *J* = 12.4, 3.5 Hz, 1H), 2.13 -2.08 (dt, *J* = 3.5, 13.2 Hz, 1H), 1.70 (td, *J* = 13.2, 2.5 Hz, 1H). C NMR (176 MHz, DMSO) δ 165.28, 161.45, 144.84, 130.03, 120.93, 113.74, 110.61, 72.14, 70.37, 69.10, 55.42, 34.78, 28.55. HRMS (ESI): exact mass calculated for C_13_H_15_NO_5_Na [(M + Na)^+^], 288.0848; found 288.0842.

**(2*S*,3*R*,4*S*,4*aR*,10*bR*)-7-Hydroxy-6-oxo-1,2,3,4,4*a*,5,6,10*b*-octahydrophenanthridine-2,3,4-triyl triacetate (20)**

Compound **17** (0.005 g, 0.012 mmol) was dissolved in 1:1 ratio of dry CH_2_Cl_2_ 0.5 mL and dry Benzene 0. 5 mL. After addition of Aluminium chloride (0.005 g, 0.037 mmol) under argon at 0 ^o^C and n-Bu_4_N^+^I^-^ (0. 0.027 g, 0.074 mmol) was added under argon slowly to the reaction mixture over a period of 10 min. The reaction mixture turned red and was stirred for 1 h at RT. After confirming full conversion with TLC, reaction mixture was quenched with water, and stirred for 2 N HCl for 0.5 h, and was extracted with EtOAc (3x10 mL). The combined organic phase was washed with brine, dried over Na_2_SO_4_, filtered through Dowex® 50WX8 hydrogen form and concentrated in vacuo and purified by flash column chromatography (CH_2_Cl_2_ / MeOH; 100:0 to 98:2) to afford compound **20** (0.0043 g, 89% Yield). [α]^23^_D_ = +45 (c = 0.5, MeOH, l = 1 dm). ^1^H NMR (700 MHz, CDCl_3_) δ 12.21 (s, 1H Ph-O*H*), 7.41 (t, *J* = 7.75 Hz, 1H, Ar-*H*), 6.91 (d, *J* = 8.4 Hz, 1H), 6.70 (d, *J* = 7.6 Hz, 1H, Ar-*H*), 5.98 (s, 1H, CON-*H*), 5.47 - 5.44 (m, 1H, C*H*-OAc), 5.23 - 5.19 (m, 2H, C*H*-OAc, C*H*-OAc), 3.84 (dd, *J* = 12.7, 10.9 Hz, 1H, C*H-*N), 3.21 (td, *J* = 12.7, 3.7 Hz, 1H, Ph-C*H*), 2.53 (d, *J* = 14.5 Hz, 1H, C*H*_2_), 2.15 – 2.14 (m, 3H, COC*H*_3_), 2.10 (d, *J* = 5.3 Hz, 3H, COC*H*_3_), 2.08 (m, 3H, COC*H*_3_), 1.97 (ddd, *J* = 15.0, 9.5, 3.1 Hz, 1H, C*H*_2_). ^13^C NMR (176 MHz, CDCl_3_) δ 170.18, 170.15, 169.37, 169.18, 162.14, 140.17, 135.01, 116.91, 113.87, 110.69, 71.84, 68.55, 67.38, 52.72, 34.62, 26.53, 21.05, 20.80, 20.71.  HRMS (ESI): exact mass calculated for C_19_H_21_NO_8_Na [(M + Na)^+^], 414.1165; found 414.11564.

**(2*S*,3*R*,4*S*,4*aR*,10*bR*)-2,3,4,7-Tetrahydroxy-1,3,4,4*a*,5,10*b*-hexahydrophenanthridin-6(2H)-one (8)**

Compound **20** (0.0043 g (0.011 mmol) and potassium carbonate (0.002 g, 0.010 mmol) were MeOH (1 mL) and stirred at RT until a white solid precipitated. TLC analysis showed full conversion after 6 h. The mixture was concentrated under a flow of N_2_. The white product was dissolved in 9:1 CH2Cl2 / MeOH and filtered through a pad of silica. The white solid was recrystallized from methanol to afford trihydroxy compound **8** (0.0026 g, 96% yield). [α]^23^_D_ = + 28 (c = 0.25, MeOH, l = 1 dm). ^1^H NMR (600 MHz, CD_3_OD) δ 8.45 (s, 1H, Ph-O*H*, 80% was deuterated), 7.29 (t, *J* = 7.81 Hz, 1H, Ar*H*), 6.70 (d, *J* = 7.7 Hz, 1H, ArH), 6.68 (d, *J* = 8.3 Hz, 1H, ArH), 4.00 (dd, *J* = 2.9, 6.3 Hz, 1H, CH_2_C*H*OH), 3.84-3.82 (m, 1H CH_2_ CHC*H*OH), 3.79 (dd, *J* = 10.3, 2.8 Hz, 1H, C*H*OH), 3.44-3.38 (dd, *J* = 10.3, 12.7 Hz, 1H, C*H*NH), 2.98 (td, *J* = 11.0, 3.69 Hz, 1H, PhC*H*), 2.20 (dt, *J* = 13.4, 3.19 Hz, 1H, C*H*_2_), 1.77 (m, 1H, C*H*_2_). ^13^C NMR (151 MHz, DMSO) δ 169.54, 160.95, 143.10, 134.27, 115.03, 113.83, 111.02, 71.69, 69.34, 68.45, 55.32, 33.75, 28.05. HRMS (ESI): exact mass calculated for C_13_H_15_NO_5_Na [(M + Na)^+^], 288.0848; found 288.0840.

1. McNulty, J., Zepeda-Velazquez, C., *Angew. Chem. Int. Ed*. **2014**, *53*, 8450- 8454.
2. Wang, L., Hubert, J. A., Lee, S. J., Pan, J., Qian, S., Reitman, M. L., Strack, A. M., Weingarth, D. T., MacNeil, D. J., Weber, A. E., Edmondson S. D., Bioorg. & Med. Chem. Lett., **2011,** *21*, 2911-2915.

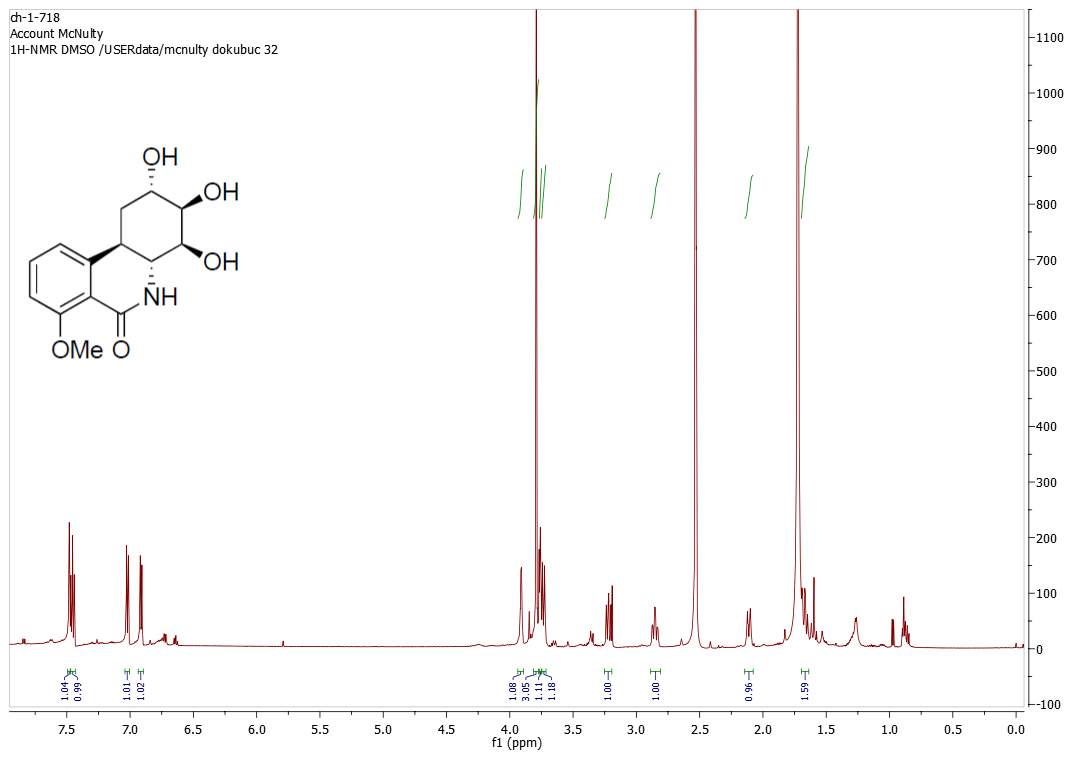


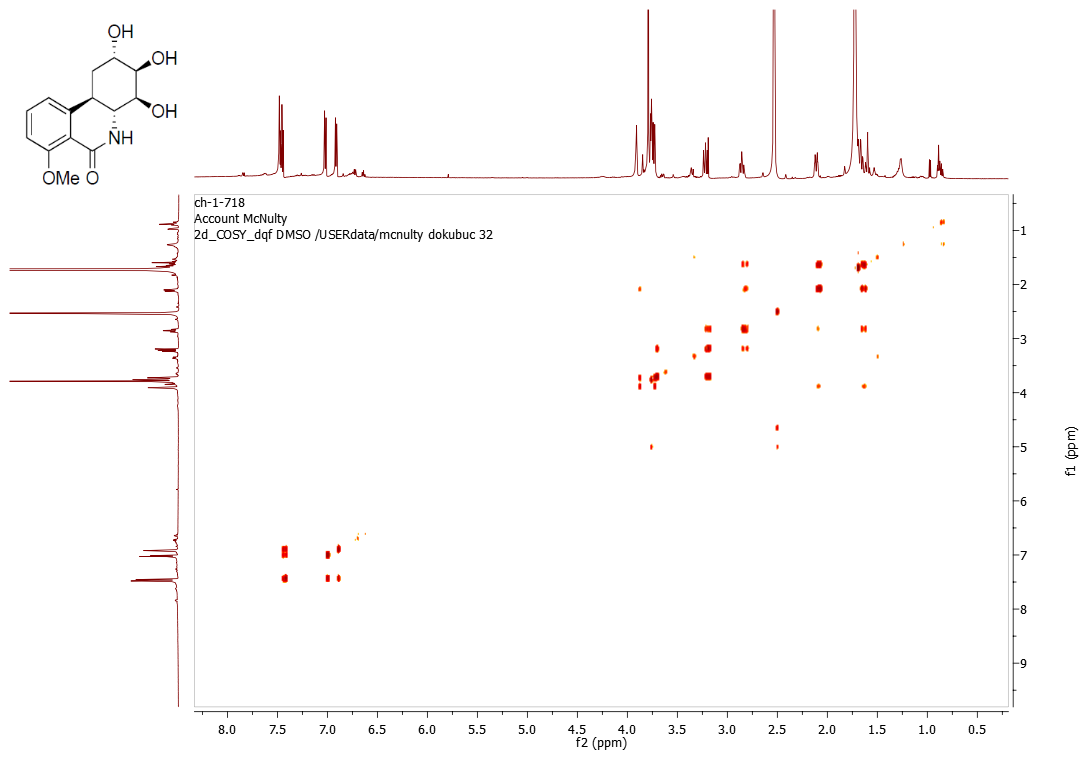


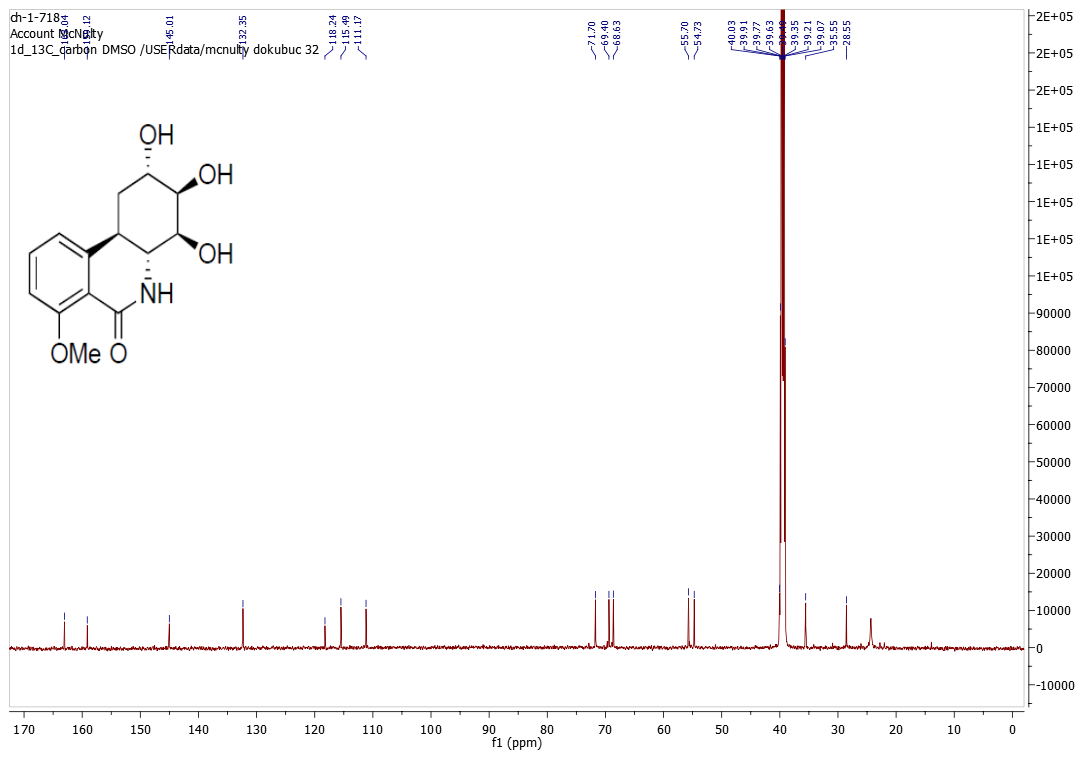

Chiral HPLC analyses.

**B) Antiviral activity assessment.**

**Generation of neural progenitor cells (NPCs).**

hiPSCs were cultured in mTeSR1-plus medium supplemented with dual SMAD inhibitors SB 431542 and LDN 193189 to promote neural induction. After 8-10 days, neural rosettes were manually isolated, transferred into Matrigel coated plates and cultured in StemDiff Neural Progenitor Medium (STEMCELL Technologies) for the expansion of NPCs.

HiPSCs were cultured in mTeSR1-plus medium supplemented with dual SMAD inhibitors SB431542 (10 µM) and LDN193189 (100 nM) (NPS/Dual-SMAD) to induce neuroectoderm formation. After 8-10 days, neural rosettes were manually isolated, transferred into Matrigel coated plates and cultured in StemDiff Neural Progenitor Medium (STEMCELL Technologies) for the expansion of NPCs.

Generation of organoids

Human iPSC (hiPSC) lines 01SD and 9001 were employed to generate brain-like organoids. Organoids were generated as previously described (ref) with some modification in the initial part regarding the generation of spheroids containing neural rosettes. hiPSCs cultured with mTeSR^TM^ plus medium (STEMCELL Technologies) in Matrigel-coated 6-well plates were detached with Accutase and then dissociated into single cell suspension by gently pipetting. They were seeded into low attachment U-bottom 96-well plates at the density of 9000 cells/well, in mTeSR plus medium supplemented with Rho-associated protein kinase inhibitor (ROCK inhibitor) Y27632 (STEMCELL^TM^) to generate embryoid bodies (EBs). After three days, to induce neuroectoderm, the medium was switched to Essential 6 medium (ThermoFisher Scientific) supplemented with dual SMAD inhibitors SB431542 10µM (MilliporeSigma S4317-5MG) and LDN193189 100nM (MilliporeSigma SML055-9-25MG. Cultures were observed on daily basis.

On day 8, differentiating EBs were rinsed with Dulbecco's Modified Eagle Medium: Nutrient Mixture F-12 (DMEM/F12, Gibco 11330-032) and cultured in neuronal medium (DMEM/F12 supplemented with 1X MEM Nonessential Amino Acid supplement (MEM-NEAA, CORNING^®^ 25-025-CI), 1X Glutamax (Gibco 35050-061), 1× N_2_ supplement (Gibco 17502-048) and 1µg/ml Heparin (STEMCELL^TM^ 07980)) in untreated 10-cm petri dishes. These plates were placed on an orbital shaker in the incubator at 70rpm (Orbi-Blotter™). Culture medium was halved every three days.

On day 20, the culture medium was replaced with cortical organoid differentiation medium I ((CODMI: DMEM:F12/Neurobasal (1:1 v/v) supplemented with 1X Glutamax, 1X B-27 (VitA[-]), 0.5X Non-essential amino acids, 0.5X N-2, Insulin (2.5 μg) and 1X penicillin-streptomycin (P/S). On day 25, CODM-I medium was replaced with cortical organoid differentiation medium II (CODM-II: DMEM/F12-Neurobasal (1:1 v/v) supplemented with 1X glutamax, 1X B-27 (VitA[+]), 0.5X Non-essential amino acids, 0.5X N-2, Insulin (2.5 μg), BDNF (10 ng/ml), and 1X P/S. Culture medium was changed every 3 days. On day 42, CODM-II medium was replaced with BrainPhys™ Neuronal medium (StemCell Technologies).

Infection of neuronal progenitor cells (NPCs).

Monolayer cultures of NPCs were infected at an MOI of 0.1 with an HSV-1 recombinant virus expressing the reporter genes EGFP and RFP under the control of the HSV-1 promoters ICP0 and gC, respectively (Ref). Two hours after the infection the inocula were removed, cells were washed and cultured in StemDiff Neural progenitor Medium supplemented with the above described R430-analogs at the concentration of 10 μM or ACV at 50 μM. Cells were analyzed by flow cytometry at day 3 p.i.

Infection of organoids.

Fourteen-week old organoids were infected singularly in U-bottom low attachment 96-well plates with an HSV-1 DualF construct (6000 pfu/organoid). After 2 hours, the inocula were removed, the organoids were washed and cultured in BrainPhys medium supplemented with the tested compounds (10 μM) or ACV (50 μM). The organoids that were infected in the presence of ACV were pretreated with the antiviral for 24 hours.

Organoids viability.

Organoids viability was analyzed using calcein AM assay (BioLegend; catalogue # 425201), according to manufacturer’s instructions. Briefly, organoids were exposed singularly in Eppendorf tube containing Calcein-AM 0.01μM and incubated at 37°C. After 20 min, organoids were transferred in prewarmed culture medium and incubated for additional 10 min. After incubation, fluorescence signals were recorded using a LEICA DMIL LED Fluorescent microscope through a 0.16NA 4x air objective.

The corrected total cell fluorescence (CTCF) was obtained by measuring EGFP fluorescence in 799-treated and untreated organoids, and normalizing for whole organoid area using equation CTCF = integrated density - (whole organoid area x mean fluorescence of background readings).

**RNA sequencing.**

NPCs were cultured in StemDiff Neural progenitor Medium, containing R430, 799, (10 µM) or vehicle. All assays were conducted in triplicate.  Cells were harvested after 72 hours and the cellular RNA was extracted (RNeasy Mini Kits, Qiagen) and quantified using Agilent 4200 TapeStation (Agilent Technologies). Total RNA libraries were generated using the Illumina TruSeq Stranded Total RNA Sample Preparation Guide, Revision E. The first step involved the removal of ribosomal and mitochondrial RNA using biotinylated, target-specific oligos combined with Ribo-Zero rRNA removal beads. Following purification, remaining RNA was fragmented using divalent cations under elevated temperature, which were then copied into first strand cDNA using reverse transcriptase and random primers, followed by second strand cDNA synthesis using DNA Polymerase I and RNase H. Subsequently, a single adenosine base was added to each of the cDNA fragments, followed by ligation of an adapter. The products were purified and enriched with PCR to create the final cDNA library. The cDNA libraries were validated using KAPA Biosystems primer premix kit with Illumina-compatible DNA primers and Qubit 2.0 fluorimeter. Quality was examined using an Agilent Bioanalyzer Tapestation 2200. The cDNA libraries were pooled at a final concentration 1.8pM. Cluster generation and 100 bp paired-read dual-indexed sequencing was performed on Illumina NextSeq 500 (Children’s Hospital of Pittsburgh, University of Pittsburgh).

Sequencing read quality was assessed using fastQC v0.11.4 and CLCbio v11.0.1 software. The average number of reads per sample was 39.5 million (SD = 4.8 million reads). Sequences were trimmed based on quality score using the modified-Mott trimming algorithm as implemented in CLC bio software, using a trim cutoff error probability of 0.05. Ambiguous bases were trimmed using a post trim maximal ambiguous base cutoff of 2. The trimmed reads were then mapped to the human genome GRCh38/hg38, using sequence and annotation provided by Ensembl (release 82). Approximately 92% of reads were mapped in pairs (SD = 1.14) across all samples, and 97.7% of reads were mapped in total (SD = 0.45). The data were deposited in NCBI’s Gene Expression Omnibus database (GSE201156).

**Quantitative RT-PCR (qRT-PCR).**

Quantitative reverse transcription PCR (qRT-PCR) was used to determine gene expression analysis using Taqman Assays (Thermo Fisher, US) specific for TP53 (ID Hs01034249_m1), SIRT1 (Hs01009006_m1) and BCL2 (Hs00608023_m1) genes and ATF4 with primers set: FWD 5’TCAAACCTCATGGGTTCTCC3’ and REV: 5’GTGTCATCCAACGTGGTCAG3’. Every sample had 3 or 4 biological replicates. Reverse transcription reaction was obtained from 100 ng of total RNA using random hexamers and SuperScript IV (Thermo Fisher). The conditions for the RT reaction were priming at 65°C for 5 min, following 55°C for 10 min and 80°C for 10 min; then held on 4°C. 2 μL of diluted cDNA (1:3) was added to Luna Universal Probe qPCR Master Mix or Luna Universal qPCR Master Mix (NBE, USA) to the total volume of 10uL. PCR was performed in CFX96 (BioRad, USA) under following conditions: 95°C for 60s, followed by 40 cycles of 95 °C for 15 s and 30 °C for 1 min. The levels of gene expression were determined using Ct (threshold cycle). The ∆Ct was calculated by subtracting the Ct of GAPDH (FWD: 5’ACCCACTCCTCCACCTTTG3’, REV: 5’CTCTTGTGCTCTTGCTGG3’) from the Ct of interest gene. ∆∆C was calculated by subtracting ∆C of the reference sample from the ∆C of the control samples. Fold change was presented by the following equation 2^-ΔΔ^ Ct.


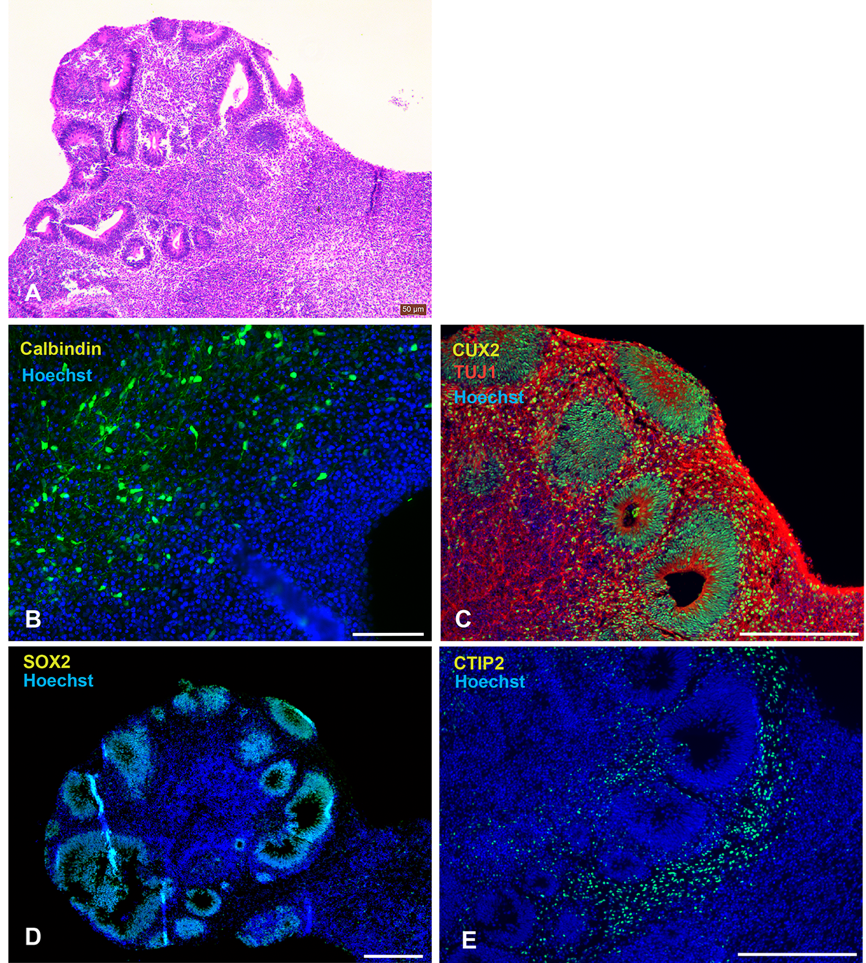


**Supplementary Figure 1. Characterization of 9001 brain organoids (A) Hematoxylin and eosin staining of 9-weeks old organoid.** (**B-E**) Immunostaining of 8 microns thick sections of organoids with antibodies recognizing CALBINDIN (**B**), CUX2/TUJ1 (**C**), SOX2 (**D**), CTIP2 (**E**). Nuclei were counterstained with Hoechst 33342. Scale bar is 50 μm in (**A**), 75 μm in (**B**), and 250 μm in (**C-E**).


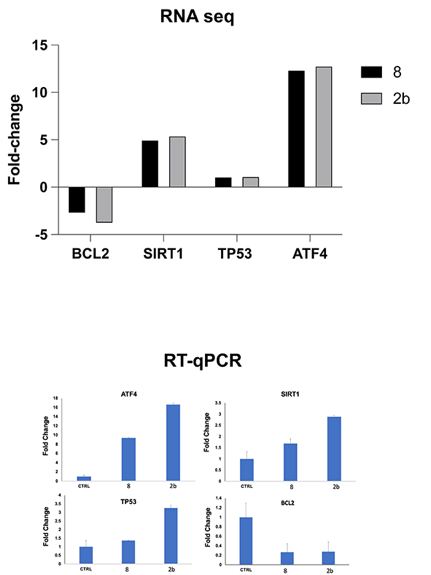


**Supplementary Figure 2. Comparison of quantitative reverse transcription PCR (RT-qPCR) and RNA sequencing assays for selected human transcripts.** RT-qPCR was used to estimate expression of four genes that showed significant **8-** and **2b**-induced changes in transcript levels in the RNA sequencing experiments. Values shown as means and error bars denote standard deviations.

**Supplementary Figure 3.** **SARSCoV-2 Antiviral Activity of Compounds 2b and 8.**

Calu3 (ATCC, HTB-55) cells were pretreated with test compounds **2b** (code R430), **8** (code CH-1-799) and remdesivir (+ control) for 2 hours prior to continuous infection with SARS-CoV-2 (isolate USA WA1/2020) at MOI=0.5. Forty-eight hours post-infection, cells were fixed, immunostained, and imaged by automated microscopy for infection (dsRNA+ cells/total cell number) and cell number. Sample well data was normalized to aggregate DMSO control wells and plotted versus drug concentration to determine the IC50 (infection: blue) and CC50 (toxicity: green).
